# Unlocking the secrets of green semiotics: The revolutionary power of eco-symbols in transforming consumer perceptions and catalyzing behavioral shifts in emerging markets

**DOI:** 10.1371/journal.pone.0310963

**Published:** 2024-09-26

**Authors:** Wongsatorn Worakittikul, Chatrawee Saenwerm, Phaninee Naruetharadhol

**Affiliations:** 1 Department of International Technology and Innovation Management, International College Khon Kaen University, Khon Kaen, Thailand; 2 Center for Sustainable Innovation and Society, International College Khon Kaen University, Khon Kaen, Thailand; 3 Business Administration Division, International College Khon Kaen University, Khon Kaen, Thailand; PLOS: Public Library of Science, UNITED KINGDOM OF GREAT BRITAIN AND NORTHERN IRELAND

## Abstract

This study delves into the intriguing dynamics between green semiotics and brand experiences, examining how elements like color, image, logo, and font not only shape brand experiences towards eco-friendly products but also influence green consumer behavior and thinking. Conducting a survey among 357 Thai consumers, this research uses Covariance-based Structural Equation Modeling (CB-SEM) to unearth the complex relationships between these semiotic elements and the multifaceted dimensions of brand experience—cognitive, sensory, emotional, and cultural. Surprisingly, the analysis revealed a predominantly negative impact of green semiotics on consumer perceptions, challenging the prevailing notion that eco-friendly branding consistently engenders positive reactions. Theoretically, this research sheds light on the potential pitfalls of green semiotics in branding, while practically, it offers critical insights for marketers on the cautious use of these elements to avoid consumer disillusionment and enhance sustainable consumer engagement, thereby contributing to a more nuanced understanding of how green brand experiences can be optimized to foster positive environmental behavior.

## 1. Introduction

Changing the world starts with a commitment to enhancing our environment, a task that is both ongoing and evolving [1]. This challenge demands more than mere awareness; it requires active engagement from individuals, communities, and nations alike [2]. In an age marked by significant environmental degradation, the quest for sustainability has shifted from a temporary trend to an essential strategy for ensuring our planet remains livable for generations to come [3]. The concept of ’green’ has evolved beyond a mere color or abstract ideal into a comprehensive movement—a rallying cry for all who are dedicated to preserving the Earth’s habitability [4]. Amidst this green revolution, aimed at reducing environmental impact, the European Union has spearheaded the initiative by establishing seventeen green objectives, influencing not only the industrial and governmental sectors but also the realms of business and education [5]. Furthermore, the rise of green production signifies a wave of continuous innovation geared towards sustainability, paralleling the growing trend of green businesses leveraging technology to turn eco-conscious ideas into tangible solutions [6]. While developed nations show a stronger link between ethical conduct and green growth, reflecting their stringent ethical standards, the commitment to pro-environmental behavior varies across regions, with Northern European countries like Sweden, Finland, and Denmark leading in eco-friendly practices [7]. Similarly, research from India highlights the significant roles of social and ethical influences on eco-friendly consumer behaviors, suggesting a targeted focus on environmental education and the use of social media to foster green purchasing discussions [8]. However, developing countries encounter obstacles in addressing eco-unfriendly practices, where traditional values, low environmental awareness, and certain lifestyles impede the progress towards Sustainable Development Goals [9]. Despite these challenges, there is momentum towards promoting green behavior, with consumer reactions to eco-friendly product indicators like ecolabels and green advertising showing a positive impact on sustainable purchasing behaviors [10]. Critical factors such as environmental concern, attitudes towards green products, and perceptions of efficacy are instrumental in shaping eco-friendly purchasing intentions and actions [11]. Hence, the global movement towards sustainability, driven by green initiatives and ethical practices, underscores the urgent need for collective action in fostering environmental awareness and promoting eco-friendly behaviors across diverse cultural and economic landscapes.

Green products are valuable in improving environmental awareness and developing a culture of sustainable living [12]. The attractiveness of eco-friendly packaging, along with a heightened feeling of eco-awareness, is rapidly affecting consumer behaviors and decision-making processes [13]. This trend is especially evident in Thailand, where there is a developing dedication to sustainable consumption and production [14, 15]. Consumers are avidly seeking eco-conscious items, frequently via internet channels, as a method to acquire sustainable alternatives [16]. Research, Bhujbal and Shafighi [17], have shown the major beneficial impacts of green packaging, eco-friendly goods, and education on trash recycling in encouraging choices that favor green transportation. Additionally, research by Jaiswal et al. [18] alludes to the favorable influence of environmental labeling and ads on eco-friendly shopping patterns. At the foundation of this green movement is the notion of green design, which is gaining importance in contemporary design for its focus on employing ecologically friendly packaging materials and processes. This coincides with the worldwide movement towards sustainable development [19]. Green design goes beyond materials, embracing the visual language of packaging—text [20], images [21], and color [22]—to successfully express the green message and fulfill consumer behavior and desires [23]. It advocates a holistic approach to product design, advocating for the integration and reconstruction of packaging with an eye on reducing environmental effects during the product’s lifespan [24]. Hence, the escalating trend towards green products and sustainable practices reflects a growing consciousness and demand among consumers for environmentally responsible choices, underscored by the critical role of green design in bridging the gap between eco-friendly intentions and consumer engagement.

Moreover, the relevance of green semiotics—symbols, signs, and messages that encompass sustainability and eco-friendliness—can be emphasized [25, 26]. Whether via product packaging or business branding tactics, green semiotics bridge the gap between green goals and customer perceptions, driving behavior towards more sustainable choices [27]. This sophisticated type of communication not only represents the values of environmental stewardship but is also firmly woven in the fabric of daily life, urging customers towards adopting more sustainable choices [28]. In essence, green design and green semiotics jointly play a crucial role in product packaging, not only in encouraging green behavior and thinking but also in creating solutions that are sustainable and visually attractive [29, 30]. Together, forming a comprehensive approach for supporting eco-friendly consumer habits and furthering the objective of sustainable development [31]. Additionally, Song et al. evaluate the impact of green semiotics on green products to assess customers’ green perceptions and the shift in green behavior and attitudes for improving eco-friendly design efficiency [32]. It further examines the implications of green semiotics for green design. In this context, the research poses a key question: How does green semiotic packaging design influence green consumer behavior and thinking through the green brand experience?

This study addresses a notable gap in the existing literature by applying semiotic knowledge in a context that has rarely been explored previously. While prior research like that conducted by [33] has demonstrated how effectively designed logos and colors consistent with a business’s strategy can influence consumer behavior using a semiotic approach, and Song et al. [34] has explored how identity, expressed through textual elements, can motivate consumers, there remains a lack of comprehensive investigation into the synergistic effects of semiotic elements such as color, images, logos, and typography. These studies often isolate rather than examine these elements in concert, particularly in their collective impact on brand experience. Additionally, although the research by [35] highlights the importance of brand experience in fostering environmental behavior intentions within marketing contexts and influencing purchasing decisions, these studies typically do not delve deeply into the various dimensions of the green brand experience. Moreover, while [36] touched upon the relationship between semiotic elements and customer experience, specific interactions between these elements and brand experience within the context of green products require further exploration. Particularly underexplored is the dimension of cultural experience within brand interactions, which is crucial for a comprehensive understanding of brand impact across different consumer segments. Though Harun et al. [37] have noted that semiotic analysis can uncover cultural messages imprinted on brands and products, there remains a paucity of research examining how cultural experience interacts with other brand experience dimensions—cognitive, sensory, and emotional. Moreover, there is a scarcity of research that considers green design from a semiotic perspective, particularly with an emphasis on eco-friendly products. Studies referenced [38–40] have begun to uncover the potential of semiotics and branding to catalyze changes in environmentally friendly consumer attitudes and behaviors. Yet, comprehensive studies that integrate these elements to reveal their collective impact on fostering sustainable consumer practices remain limited. To address these gaps by providing detailed insights into how semiotic elements can be strategically employed to enhance all dimensions of the green brand experience—cognitive, sensory, emotional, and cultural—and thereby promote environmentally responsible behavior among consumers. Thus, this research will unlock the secrets of semiotic elements and brand experience dimensions, revealing their profound impact on consumer perceptions and behaviors towards eco-friendly products.

To address these gaps, this current research has chosen to investigate the interplay between elements of green semiotic packaging and dimensions of green brand experience, exploring how this relationship influences green behavior and thinking associated with green products. To address the research question, "How do green semiotic elements influence green consumer behavior and thinking through the green brand experience?", this research aims to investigate the relationship between the elements of green semiotics and dimensions of green brand experience and their impact on the transformation of consumer attitudes and behaviors towards green products.

Thailand serves as the case study setting for examining green products within an emerging market. This study advances the theoretical framework connecting the semiotics approach with brand experience, which are critical in shaping customer attitudes and behaviors towards green products. As far as the authors are aware, this is the inaugural study to explore the link between green semiotic elements in product design and dimensions of green brand experience, as well as their effect on modifying consumer attitudes and behaviors towards green products. This study makes significant contributions to green marketing and sustainable business strategies. It extends the theoretical framework of semiotics to the marketing of eco-friendly products, offering a comprehensive analysis of how elements such as colors, logos, images, and fonts shape consumer behavior and brand perception. Furthermore, empirical results from structural equation modeling illustrate the effects of these green semiotic elements on different facets of brand experience—cognitive, sensory, emotional, and cultural—within environmentally friendly products. Additional analyses underscore the specific impacts of these semiotic strategies, demonstrating their varied influences on the design aspects of green brand experiences and how these differences shape consumer perceptions and behaviors towards sustainable products.

The paper features a well-defined structure. Section 2 presents a comprehensive analysis of the existing literature and outlines the specific parameters chosen for incorporation into the model. In the following section, we elucidate the research strategy and methodology employed, utilizing data collected from 357 environmentally conscious participants in Thailand. Section 4 contains the empirical results and findings. Argumentative discussions and implications are discussed in Section 5. In conclusion, Section 6 addresses the paper’s conclusion, the limitations, and suggests future directions for the study.

## 2. Review of literature

### 2.1 Theoretical background

#### 2.1.1 Green products in emerging market

Green products are more important in developing countries than in developed nations due to reasons including fast industrialization, urbanization, and growing environmental concerns [7]. Aspecaily, Green products in the emerging market of Thailand are gaining attention from consumers who are increasingly concerned about sustainability and the environment [14, 15]. The shift towards sustainable consumption in Thailand has also involved companies in the appliance and dairy industries, with a focus on sustainable supply and consumption processes [41]. Green products, which are eco-friendly or environmentally friendly goods or services, are becoming more popular in developing countries due to increasing environmental consciousness and a rising demand for sustainable options among customers [42]. Businesses in various sectors are using environmental lobbying to push for more eco-friendly legislation, emphasizing the significance of sustainable practices and goods [43]. Businesses that focus on eco-friendly products are engaging in environmental lobbying to advocate for incentives and subsidies, acknowledging the advantages of supporting sustainability efforts [44]. Developing environmentally friendly goods in developing countries has distinct hurdles, such as costly research and development and restricted availability of ecologically advantageous technology like biodegradable polymers and recycled materials [45]. Many organizations are overcoming hurdles by using new marketing methods like semiotics to boost market share for eco-friendly goods [46]. This strategy focuses on creating items that clearly convey the brand’s identity, increase customer recognition, and boost the brand’s reputation [47]. Effective communication in this process relies heavily on semiotic design, which requires a thorough understanding of language, symbols, and culture [48]. Moreover, companies may influence customer buying choices and develop confidence in their brands by effectively using symbols [49]. Therefore, a comprehensive analysis of semiotics in design is crucial for ensuring clear communication and promoting the acceptance of eco-friendly goods in developing markets.

#### 2.1.2 Semiotics in green products or green semiotics

Semiotics, the study of signs and symbols, is often used in corporate communication to transmit information efficiently, was definited by [50]. The semiotics approach helps marketers understand the relationship between signs and the messages they convey, giving them better control over the communication process [51]. Recently, green marketing has used semiotics to convey the environmentally friendly features of goods to customers in light of the increasing significance of environmental sustainability in business [52]. Semiotics improve brand communication and impact customers’ subconscious decision-making via systematic, reliable, and culturally significant signals, including *color*, *font*, *picture*, *and logo* [40, 53]. These aspects are essential for communicating messages and influencing views [54]. Color selection may elicit certain affective experiences [55], while font type can communicate the brand’s tone or personality recognition [34]. Utilizing the logo and picture in marketing materials strengthens brand identification and graphically conveys the product’s characteristics [56, 57]. A well-crafted design effectively communicates the brand’s message and values concisely [58]. Semiotics differentiates between persuasion and manipulation in commercial communication, enabling marketers to create convincing yet ethical message tactics [25]. Semiotic analysis is useful for revealing business model information in corporate reports, helping companies maintain their legitimacy and regulatory compliance [28]. Semiotics continue to be essential in commercial communication in the digital age, especially as symbols and icons become more important in visual communication [59]. Thus, Semiotics is a crucial instrument in corporate communication, facilitating successful message delivery, brand management, legitimacy management, and regulatory compliance.

#### 2.1.3 Green brand experience

Green brand experience contains the words “green” and “brand experience". The first word refers to the green concept, which is a set of principles and practices focused on advancing environmental sustainability and reducing damage to the world [60]. And the second word is mentioned as referring to a situation in which customers make specific cognitive, emotional, sensory, and behavioral investments to receive a valuable experience from interacting with a brand [61]. Evidently, the green brand experience refers to the consumer’s perception and interaction with a brand that is environmentally friendly [35]. Green behavior is influenced by various factors, such as interactivity and green informativeness, in social media marketing management activities [62]. Other determinants of the green brand experience include image, logo, value, satisfaction, and design [40, 63]. From previous studies, the dimensions of brand experience include thought *cognitive experience*, *emotional experience*, *sensory experience*, *and cultural experience*, which are heavily used in the tourism industry, including the development of media such as advertising. Brand experiences are important in motivating and building trust, purchasing decisions, and loyalty [61]. Additionally, brand-related stimuli like design, identity, packaging, communications, and environments can influence brand experiences in advertising [55]. Color stimuli can significantly impact sensory experiences in packaging [64–66]. The use of color in packaging design can influence consumer responses at the sensory, affective, intellectual, and behavioral levels [67]. Affective or emotional experience is the band of experience dimensions that refers to personal feelings that individuals have in response to various stimuli or situations [68]. Emotional experiences can be influenced by stimuli, such as visual or auditory stimuli, as well as cognitive processes [69]. In the same way, cognitive experience refers to the conscious cognitive and emotional experiences that accompany cognitive activities [70]. Cognitive experience has been shown to be closely associated with consumer behavior research streams such as advertising and product design [71]. Cultural behavior experience in design packaging involves integrating cultural elements and consumer behavior into the packaging design process to create a meaningful and enjoyable user experience [72]. Moreover, Shukla et al. [40] have shown that semiotic product packaging has a beneficial impact on consumer brand exp erience, brand trust, and purchase intention. Thus, implementing green brand experience involves integrating sustainability principles with consumer perceptions, including factors like social media engagement and design, while semiotic packaging enhances brand trust and customer behavior.

#### 2.1.4 Shifted in Green Behavior and Thinking (SGBT)

**Fig 1:**
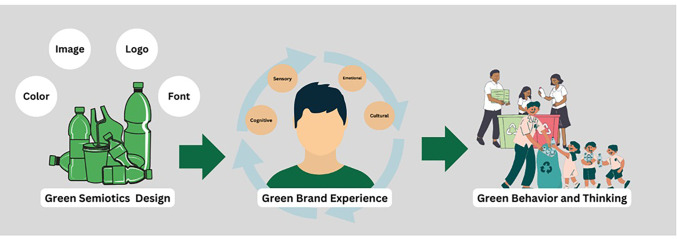
An illustration depicting the influence of semiotic elements on brand experience and their subsequent impact on fostering green behavior and thinking: Authors, 2024. “Reprinted from [https://drive.google.com/file/d/1DmkeDnDWU-4zVwI96hFQEwxW9Enwima9/view?usp=share_link] under a CC BY license, with permission from Plos One, original copyright [2024]”.

SGBT describes the shift in people’s attitudes, beliefs, and behaviors towards environmental sustainability and eco-consciousness, leading to the adoption of more ecologically friendly activities such as 3Rs (i.e., recycle, reduce, reuse).[42, 73]. However, there are some factor which is sfiftable SGBT. The study of Wu et al. [74] illustrate that green brand experience significantly influences customers’ pro-environmental behavioral intentions towards green products. Green brand experience, green brand experiential quality, and green brand cognitive dissonance impact green brand experiential pleasure, which then influences shifing intentions and behavior [75]. Perceived brand innovativeness and green image have a favorable impact on several aspects of brand experiences, such as sensory, emotional, cognitive, and behavioral experiences [76]. These characteristics partly moderate the connection between them and brand satisfaction [77]. Brand Image, trust, value, satisfaction, and loyalty are factors that affect green brand equity, with less emphasis on things like quality, awareness, characteristics, and promotional activities [78, 79]. Brand experience significantly influences customers’ environmental attitudes and actions, underscoring the need to strategically manage brand experiences to improve sustainability initiatives [78]. Moreover, Consumers who recognized eco-labelled on green items showed more pleasant emotions and were more inclined to participate in pro-environmental behaviors, including recycling [80]. The eco-label design impacts customers’ green action by enhancing their understanding and awareness of the importance of sustainability in goods [24]. Noticably, Semiotics has played a significant role in shifting green thinking and behavior by utilizing as a tool to investigate the processes of significance generated by individuals in their interaction with the environment [81]. More than that, Packaging designers should consider the visual appearance of packages as it has a significant impact on consumer behavior during purchasing decisions and sorting waste after use [82]. Therefore, strategic management of green brand experiences is vital for fostering sustainability, while eco-labels and semiotics play key roles in shaping green actions, underscoring the importance of packaging design for influencing consumer behavior as shown in Fig 1.

### 2.2 Hypothesis development

#### 2.2.1 Color in semiotic awareness of green product and green brand experience dimensions

*Relationship between color in semiotic awareness of green product and cognitive experience*. Color plays a pivotal role in the semiotic interpretation of green products, not only influencing consumer cognitive processes but also evoking a spectrum of emotional and sensory responses that enhance engagement with environmentally conscious goods. In the realm of green marketing, color functions as a powerful communication tool, capturing attention and shaping consumer perceptions, particularly through the symbolic use of green which is universally associated with sustainability and environmental responsibility [83]. This association helps to foster a deeper connection between the product and eco-conscious behavior, promoting awareness and encouraging conservation efforts [84]. Moreover, the strategic selection of colors based on cultural preferences can significantly enhance the attractiveness of products and services, reinforcing the link between visual cues and consumer cognitive experiences [22, 85]. This nuanced understanding of color’s role in environmental marketing suggests that it not only triggers direct cognitive recognition of green attributes but also subtly influences consumer behavior by reinforcing the environmental ethos a brand represents [27, 55]. Thus, it is hypothesized that there is a positive relationship between the use of color in semiotic awareness of green products and enhanced cognitive experience, as color effectively communicates and reinforces environmental values, leading to increased consumer engagement and sustainable consumption behaviors.

*H1a*: *Color in semiotic awareness of green product has positive impact on the cognitive Experience*.

*Relationship between color in semiotic awareness of green product and sensory experience*. While research directly exploring the impact of color on the sensory experience of green products remains scant, existing studies provide a foundational understanding of how color influences sensory perception in environmental contexts. Prior research indicates that sensory elements such as taste, smell, and touch, though less verbally expressible, are significantly enhanced by visual stimuli like color, which serves as a strong trigger for sensory engagement [55]. Moreover, studies suggest that the integration of visual elements can activate complex mental representations and perceptions across multiple sensory domains, including auditory, gustatory, olfactory, and tactile senses [86]. In particular, the tactile sense can be enriched through the visual perception of color, which may enhance the creative interpretation of product attributes and foster a deeper sensory connection with the product [87]. These findings underscore the multifaceted role of color in shaping sensory experiences, suggesting that color used in the semiotic design of green products can profoundly influence the sensory engagement of consumers by creating more vivid and memorable sensory impressions [63, 82]. Building on this groundwork, it is hypothesized that the color of semiotic awareness in green products positively correlates with an enriched sensory experience, as color not only visually communicates sustainability but also amplifies the sensory appeal of the products, thus enhancing overall consumer perception and interaction.

*H1b*: *Color in Semiotic awareness of green product has positive impact on the sensory experience*.

*Relationship between color in semiotic awareness of green product and emotional experience*. Research into the emotional impact of color in the context of green products reveals a complex interplay between visual cues and emotional responses. Studies have demonstrated that the design and color of environmental awareness campaigns can significantly affect public consciousness about environmental conservation, eliciting strong emotional reactions that can influence both perception and behavior [27]. Additionally, the Green Emotion Model (GEM) 2.0 suggests that environmental attitudes, heightened by effective semiotic cues like color, play a crucial role in driving consumers towards products with eco-labels [88]. This model emphasizes that appropriately chosen colors can capture attention, generate positive emotions, and motivate the purchase of eco-friendly products [24]. Furthermore, color analysis in product design has been identified as vital in forging an emotional connection with consumers, by resonating with their feelings and enhancing the emotional appeal of the products [89]. While the direct link between color and emotional experiences in the context of green products is still underexplored, the existing evidence suggests that color can significantly enhance the emotional experience by evoking feelings that align with the eco-conscious values represented by the product. Therefore, it is hypothesized that color, as a component of semiotic awareness in green products, positively influences emotional experiences, leveraging visual appeal to deepen emotional engagement and support sustainable consumer choices.

*H1c*: *Color in semiotic awareness of green product has positive impact on the emotional experience*.

**Fig 2:**
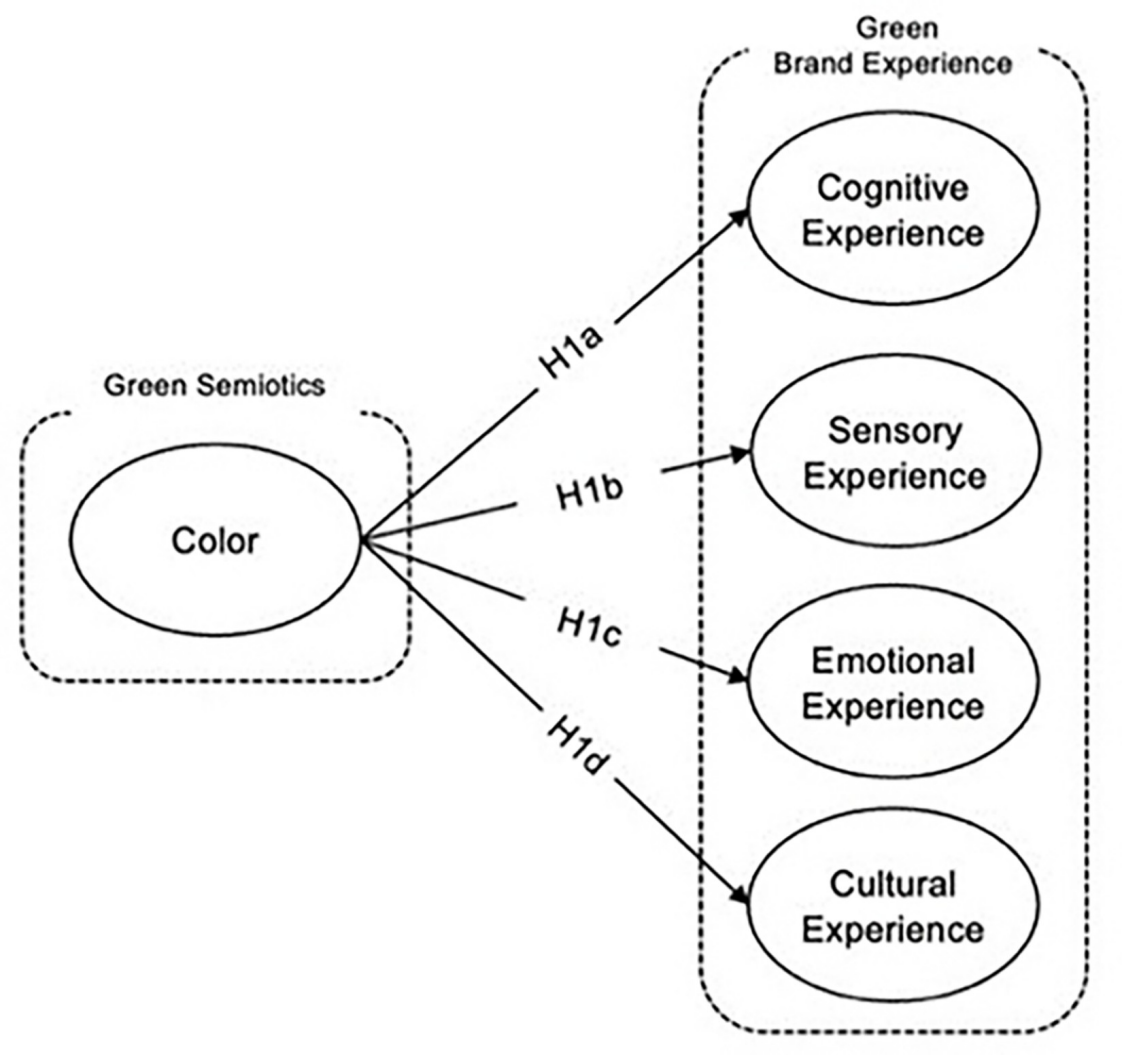
Color in semiotic awareness of green product and green brand experience dimensions hypothesis development: Authors, 2024.

*Relationship between color in semiotic awareness of green product and cultural experience*. The interplay between color and semiotic awareness of green products profoundly influences cultural experience, underlining the strategic importance of color in environmental branding. Color serves as an essential communicative tool, with its alignment to cultural preferences significantly bolstering the appeal of products and services within specific cultural contexts [56]. In the realm of green marketing, distinctive color trademarks are crucial not only for enhancing brand recognition but also for differentiating companies within the competitive landscape of green branding [49]. Furthermore, color plays a pivotal role in enhancing public awareness of green products and promoting responsible consumption patterns, which are critical for advancing environmental sustainability [90]. Cultural insights into color usage are integral to effective product design and corporate social responsibility, influencing consumer perceptions and engagement with the brand [31]. Research in semiotics further highlights that strategic use of colors, alongside other non-human elements in advertisements, can significantly increase consumer engagement with green products by resonating with cultural narratives and values [91]. Given this backdrop, it is hypothesized that the deliberate integration of culturally resonant colors in the marketing of green products enhances cultural experience, thereby strengthening consumer connection and fostering sustainable consumption behaviors illustrated in Fig 2 below.

*H1d*: *Color in semiotic awareness of green product has positive impact on the emotional experience*.

#### 2.2.2 Image in semiotic awareness of green product and green brand experience dimensions

*Relationship between image in semiotic awareness of green product and cognitive experience*. Research exploring the relationship between the image of green products and cognitive experience has uncovered significant findings that underscore the influence of visual elements on consumer perceptions and attitudes. Studies indicate that the alignment of image proximity with product type can significantly affect both advertising and product attitudes by influencing consumers’ mental imagery, thereby enhancing their cognitive engagement with the product [27]. Additionally, studies have examined how environmental experiences modify the relationship between individuals’ cognitive styles and their creative outcomes, particularly in contexts such as design education where visual cues play a pivotal role [92]. Furthermore, research incorporating Social Cognitive Theory has delved into the organizational cognition of green technologies, revealing how visual representations of these technologies can influence behavioral intentions towards environmental responsibility [93]. Based on these insights, it is hypothesized that the visual representation of green products—particularly how images are used to signify eco-friendliness—positively impacts cognitive experience by enhancing consumers’ awareness and understanding of the product’s environmental benefits, thereby fostering a more informed and engaged consumer base.

*H2a*: *Image in semiotic awareness of green product has positive impact on the cognitive experience*.

*Relationship between image in semiotic awareness of green product and sensory experience*. The relationship between the imagery of environmentally friendly products and sensory experience is pivotal in shaping consumer perceptions. While taste, smell, and texture are often less readily articulated verbally, visual imagery serves as a powerful conduit for expressing and enhancing these sensory aspects [66]. Visual representations not only help in examining societal perceptions of environmental impacts but also significantly enhance the aesthetic appeal of eco-friendly products, making them more attractive to consumers [94]. Techniques such as image elicitation and itinerant soliloquy are instrumental in delving deeper into the environmental narratives, revealing the nuanced and context-specific nature of environmental issues and how they are perceived [95]. By integrating visual and sensory strategies, researchers, practitioners, and policymakers can gain a more comprehensive understanding of the ways in which visual and sensory elements influence consumer perceptions and experiences of green products. These insights suggest that the strategic use of imagery in marketing eco-friendly products can effectively engage sensory experiences, thereby enhancing consumer engagement and promoting a deeper connection to environmental sustainability. Building on these findings, it is hypothesized that images associated with green products positively influence sensory experiences, facilitating a stronger and more meaningful consumer interaction with these products.

*H2b*: *Image in Semiotic awareness of green product has positive impact on the Sensory Experience*.

*Relationship between image in semiotic awareness of green product and emotional experience*. The connection between the imagery of environmentally friendly products and consumer emotional responses has been substantiated through various studies, indicating that visual elements can significantly affect the emotional engagement with green products. Research suggests that images depicting natural environments in the context of green products can evoke emotional experiences akin to those experienced directly in nature, thereby enhancing the emotional connection consumers have with these products [88]. Additionally, integrating product metaphors with emotionally durable design has been shown to effectively raise awareness of sustainable development issues, as products that evoke strong emotional experiences through visual cues are more likely to be cherished and retained longer by consumers [96]. Further studies have examined the emotional associations consumers make with bio-based materials, finding distinct emotional values attributed to different materials, such as sugar cane and starch-based materials being perceived as eco-friendly, and wood, paper, and palm leaf as natural [97]. The finding of [98] demonstrated that the visual representation of green products plays a crucial role in influencing consumer emotional responses, suggesting that strategically designed images can foster deeper emotional connections with the product. This enhanced emotional engagement can lead to greater consumer attachment and prolonged product lifecycles, ultimately supporting sustainability goals. Thus, it is hypothesized that the use of emotionally resonant imagery in the marketing of green products positively impacts emotional experiences, fostering stronger consumer bonds and promoting sustained interest in environmentally friendly choices.

*H2c*: *Image in semiotic awareness of green product has positive impact on the emotional experience*.

**Fig 3:**
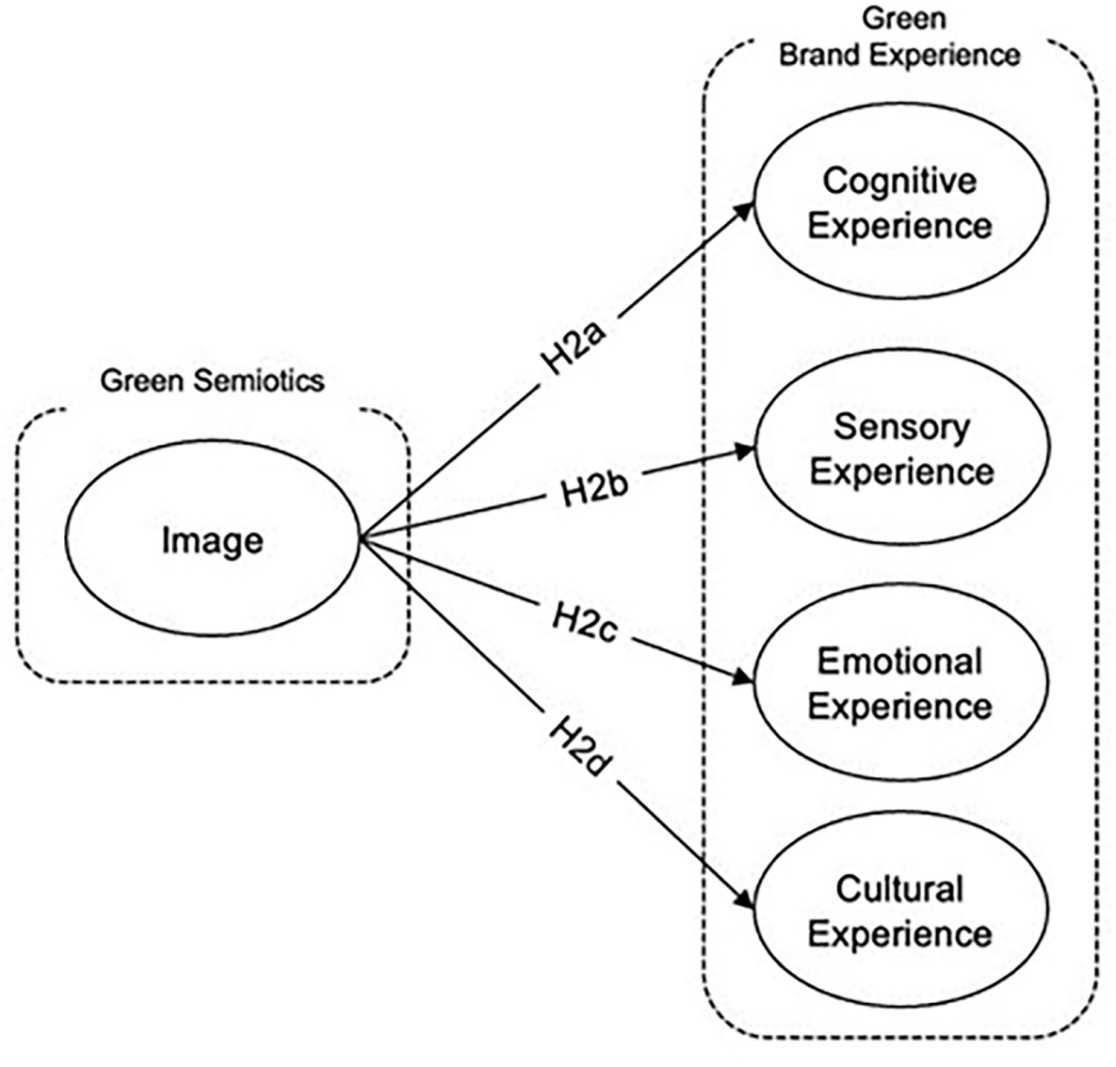
Image in semiotic awareness of green product and green brand experience dimensions hypothesis development: Authors, 2024.

*Relationship between image in semiotic awareness of green product and cultural experience*. Visual representation plays a pivotal role in shaping the cultural experience associated with eco-friendly products, significantly influencing consumer perceptions across various cultural contexts. Environmental themes, often integrated into consumer culture through mediums like greeting cards and virtual reality, facilitate connections between individuals and the natural environment, embedding sustainable values into everyday cultural interactions [99]. Furthermore, the design elements of eco-labels, such as size, saliency, shape, symbols, and language, directly impact visual attention and guide consumer decisions, swaying inclinations towards sustainable food choices [100]. The combination of visual and linguistic elements in green marketing campaigns profoundly affects how consumers perceive the eco-friendliness of a product, enhancing or diminishing their cultural resonance with the brand [91]. However, while attractive visuals can draw attention to a product’s environmental claims, they can also skew consumer perceptions, potentially overshadowing the objective evaluation of the product’s true environmental impact [96]. Therefore, it is hypothesized that effective visual representation in the semiotics of green products enriches the cultural experience, reinforcing eco-friendly perceptions and encouraging culturally informed consumer choices, ultimately fostering a deeper commitment to sustainability within various cultural frameworks. The mentioned relationship can be seen in Fig 3.

*H2d*: *Image in semiotic awareness of green product has positive impact on the cultural experience*.

#### 2.2.3 Logo in semiotic awareness of green product and green brand experience dimensions

**Fig 4:**
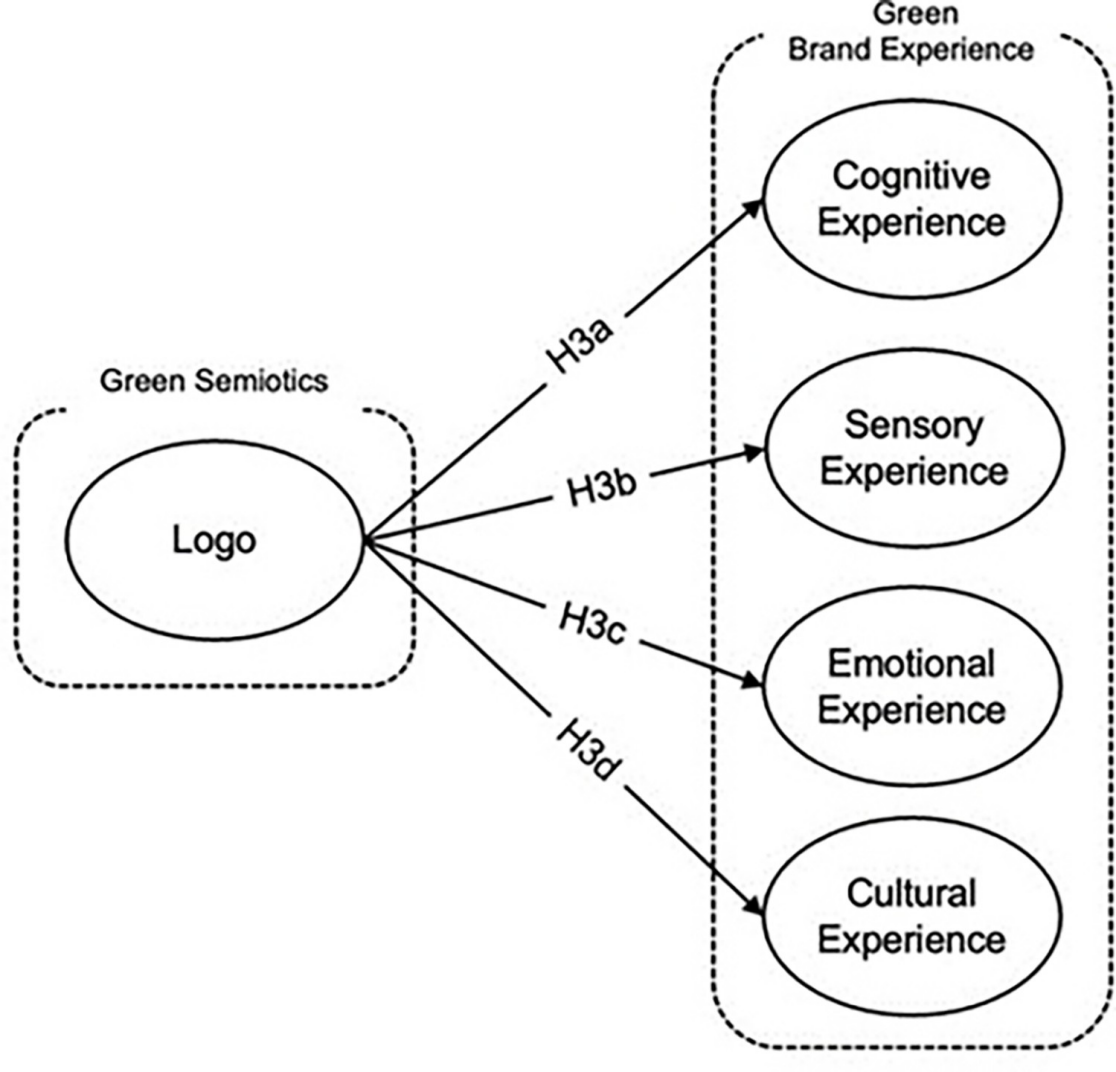
Logo in semiotic awareness of green product and green brand experience dimensions hypothesis development: Authors, 2024.

*Relationship between logo in semiotic awareness of green product and cognitive experience*. Logo representation plays a pivotal role in shaping cognitive perceptions of environmentally friendly products, serving as a key semiotic element that influences consumer understanding and recognition. Research indicates that logos incorporating green, eco-friendly hues not only signal a retailer’s commitment to environmental initiatives but also foster a favorable perception among consumers [100]. The specific use of color in logos can effectively differentiate a brand, preventing competitors from using similar color schemes that are vital for brand identification and association [49]. Additionally, the recognition and familiarity of logos are crucial for promoting green companies, as they enhance consumer awareness and knowledge of eco-friendly products [101]. The cognitive impact of logos, therefore, is substantial, as they act as visual cues that communicate and reinforce the environmental ethos of a product [102]. Given these dynamics, it is hypothesized that effective logo design in the semiotics of green products positively influences cognitive experiences, enhancing consumer awareness and promoting a stronger cognitive connection with the brand’s environmental efforts as presented in Fig 4.

*H3a*: *Logo in semiotic awareness of green product has positive impact on the cognitive experience*.

*Relationship between logo in semiotic awareness of green product and sensory experience*. Logo design plays a crucial role in shaping sensory perceptions of eco-friendly products, with its influence extending across different cultures. The color of a logo, for instance, can significantly affect consumer perceptions of a retailer’s commitment to environmental sustainability. For example, logos that incorporate a prominently eco-friendly color like green can enhance the perception of a business’s environmental consciousness, whereas logos with less eco-friendly colors such as red may suggest lesser environmental commitment [49, 103]. Additionally, research has indicated that the shape of a logo also impacts sensory exp eriences; circular logos, associated with harmony and continuity, are found to be more effective in promoting environmentally friendly consumption behaviors than angular logos, which may appear more aggressive and less conducive to sustainability messages [104]. Furthermore, the sensory appeal of materials used in product design, including those represented in logos, plays a vital role in both consumer satisfaction and environmental acceptance [105]. Given these insights, it is hypothesized that the design elements of a logo—color and shape—transfer positively towards sensory experiences, enhancing the overall perception of the product’s eco-friendliness and aligning consumer sensory responses with sustainable practices.

*H3b*: *Logo in semiotic awareness of green product has positive impact on the sensory experience*.

*Relationship between logo in semiotic awareness of green product and emotional experience*. Logo design profoundly influences the emotional experience associated with environmentally friendly products across various cultural contexts. The design of a brand’s logo can act as a powerful catalyst, evoking emotional responses that reflect consumer beliefs about the brand’s environmental commitment [56]. These emotional responses are critical as they can significantly influence consumer behaviors, including their purchasing decisions. A well-designed logo that resonates emotionally can enhance the perception of a brand’s dedication to sustainability, leading to increased consumer trust and loyalty [102]. Positive emotional connections facilitated by such logos can motivate consumers to make responsible purchasing decisions and opt for products that are both ecologically and socially responsible [88]. Previous research supports this by showing that emotional engagement driven by effective logo design can contribute significantly to green consumption practices and offer practical benefits for companies marketing green products [106]. Therefore, it is hypothesized that logo design in green product semiotics plays a crucial role in shaping emotional experiences, with effectively crafted logos enhancing the emotional appeal of a brand’s commitment to environmental sustainability.

*H3c*: *Logo in semiotic awareness of green product has positive impact on the emotional experience*.

*Relationship between logo in semiotic awareness of green product and cultural experience*. Logo design plays a pivotal role in shaping cultural perceptions of eco-friendly products, with its effectiveness heavily influenced by the cultural context and the symbolic use of color. Colors used in logos not only communicate specific messages but also capture attention, making them essential tools for branding in diverse cultural settings [49]. In societies with rich cultural traditions, such as India, where the concept of unity in diversity prevails, the choice of colors and design elements in logos can significantly affect consumer acceptance and brand differentiation. Distinctive logos that incorporate culturally resonant colors and themes help in safeguarding brand identity against competitors, enhancing brand recognition [107]. Additionally, pro-environmental values such as environmental awareness and concern are crucial in shaping how logos are perceived within different cultures. These values influence consumer attitudes towards brands that effectively integrate environmental themes into their logo designs [75, 100]. Thus, for logos to be effective in communicating the eco-friendliness of products across various cultures, they must align with both local cultural values and global environmental concerns. It is hypothesized that culturally informed logo designs, which resonate with pro-environmental values, significantly enhance the cultural experience of eco-friendly products, fostering greater consumer engagement and brand loyalty.

*H3d*: *Logo in Semiotic awareness of green product has positive impact on the cultural experience*.

#### 2.2.4 Font in semiotic awareness of green product and green brand experience dimensions

*Relationship between font in semiotic awareness of green product and cognitive experience*. Font design is a crucial element in semiotics that significantly influences the cognitive experience associated with eco-friendly products. The selection of fonts in environmental messaging can evoke specific feelings and perceptions, which in turn shape consumer attitudes towards sustainability [108]. Aesthetically appealing typefaces not only elicit positive emotional responses but also enhance the overall perception of a product’s environmental friendliness [96]. Additionally, the strategic use of font design can facilitate cognitive engagement by helping consumers better understand the context and intricacies of environmental issues. This is achieved by supporting relational perceptual processes that elucidate the relationships and causative factors underlying environmental challenges [109]. Understanding consumer preferences in font design is essential for designers aiming to create impactful and appealing communication materials [110]. These materials can significantly boost both emotional and cognitive engagement with eco-friendly products, promoting a deeper understanding and appreciation of their environmental benefits [13]. Therefore, it is hypothesized that the thoughtful application of font design in green product semiotics positively influences cognitive experiences, enhancing consumer awareness and encouraging more informed decisions regarding sustainable consumption.

*H4a*: *Font in semiotic awareness of green product has positive impact on the cognitive experience*.

*Relationship between font in semiotic awareness of green product and sensory experience*. Font design significantly influences sensory perception and enhances the appeal of eco-friendly products by imparting a unique "personality" to the materials used. The strategic use of typeface design not only boosts the visual appeal of these products but also facilitates their wider adoption by making them more aesthetically appealing [111]. Beyond mere aesthetics, font design plays a pivotal role in sensory marketing, where it is utilized to enhance user-product interaction and to evoke positive sensory responses that are critical for product acceptance [110]. This interaction is especially significant as it affects cross-cultural sensory modalities and emotional reactions, contributing to an overall favorable user experience. In various industries, typeface design is a key component of sensory marketing strategies aimed at strengthening the emotional connection between consumers and brands [112]. Given these insights, it is hypothesized that font design, when effectively integrated into the semiotics o f eco-friendly products, can significantly enhance sensory experiences, thereby promoting a deeper emotional and sensory engagement with products that embody environmental sustainability.

*H4b*: *Font in semiotic awareness of green product has positive impact on the sensory experience*.

*Relationship between font in semiotic awareness of green product and emotional experience*. Although existing literature has only tangentially touched upon the relationship between typeface design and emotional responses to eco-friendly products, emerging research is beginning to highlight its significance. Wang et al. [88] demonstrate how the presentation of resource usage information, influenced by typeface design, can modify user perceptions and behaviors, enhancing the product’s appeal and its effectiveness in promoting sustainable practices. This research supports the idea that the emotional impact of design elements, including fonts, can significantly augment a product’s attractiveness and perceived efficacy. Furthermore, Ji et al. [113] provide evidence that typeface design, as a critical visual element, can amplify the emotional impact of eco-friendly product designs, thereby influencing consumer reactions and engagement. Ho’s research [114] also underscores the importance of typography in shaping communication and narrative, suggesting that the choice of typeface can create a strong connection between the visual design elements and the textual messages, enhancing the emotional resonance of the communication. Building on these insights, it is hypothesized that typeface design in green product semiotics plays a crucial role in enhancing emotional experiences, by effectively conveying eco-friendly values and eliciting positive emotional reactions that can lead to increased consumer attachment and sustained interest in environmentally responsible products.

*H4c*: *Font in semiotic awareness of green product has positive impact on the emotional experience*.

**Fig 5:**
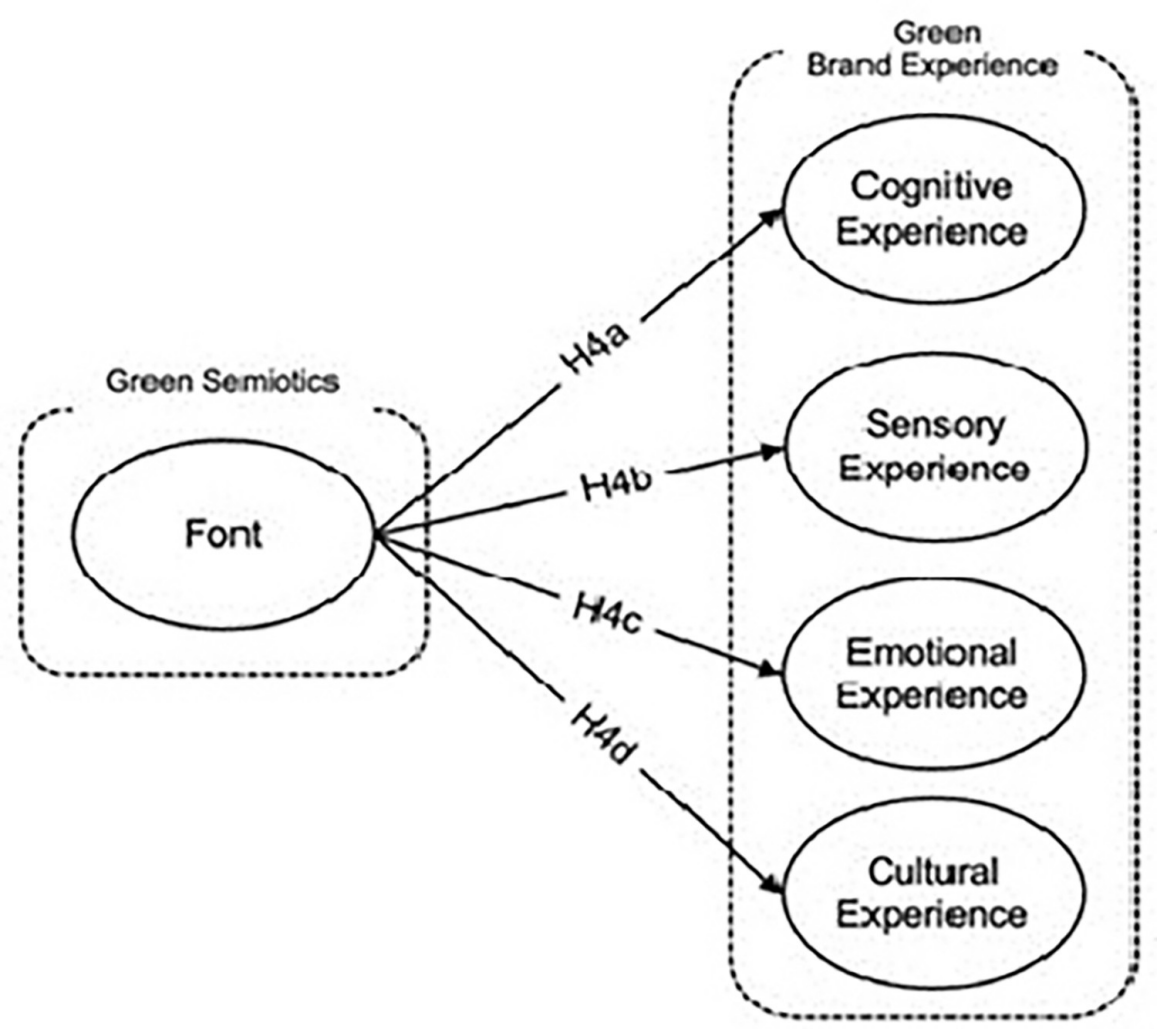
Font in semiotic awareness of green product and green brand experience dimensions hypothesis development: Authors, 2024.

*Relationship between font in semiotic awareness of green product and cultural experience*. The relationship between font design and the cultural experience associated with eco-friendly products in different cultures is nuanced and underexplored. Research into the aesthetics of digital fonts highlights the importance of considering users’ creative experiences within digital environments, suggesting that font design can play a significant role in enhancing the user interface by accommodating cultural preferences [110]. Further studies underscore the impact of a user’s cultural background on their interaction with design elements such as typefaces, pointing to the necessity of incorporating cultural diversity into design practices to optimize user engagement [54]. Cultural values are known to profoundly influence the design of environmentally friendly products, impacting everything from material choices to color schemes and typographic details [99]. These insights suggest that understanding cultural sensitivities is essential for designers aiming to create eco-friendly products that resonate with diverse consumer bases. Thus, it is hypothesized that font design, when carefully aligned with cultural experiences and preferences, significantly influences the cultural reception of green products. Effective typography can bridge cultural gaps, facilitating a deeper connection between consumers and the environmental values the products represent, thereby enhancing the overall appeal and acceptance of eco-friendly goods. These relationships can be found in Fig 5.

*H4d*: *Font in semiotic awareness of green product has positive impact on the cultural experience*.

#### 2.2.5 Green Brand Experience (GBE) dementions and Shift in Green Thinking and Behavior (SGTB)

**Fig 6:**
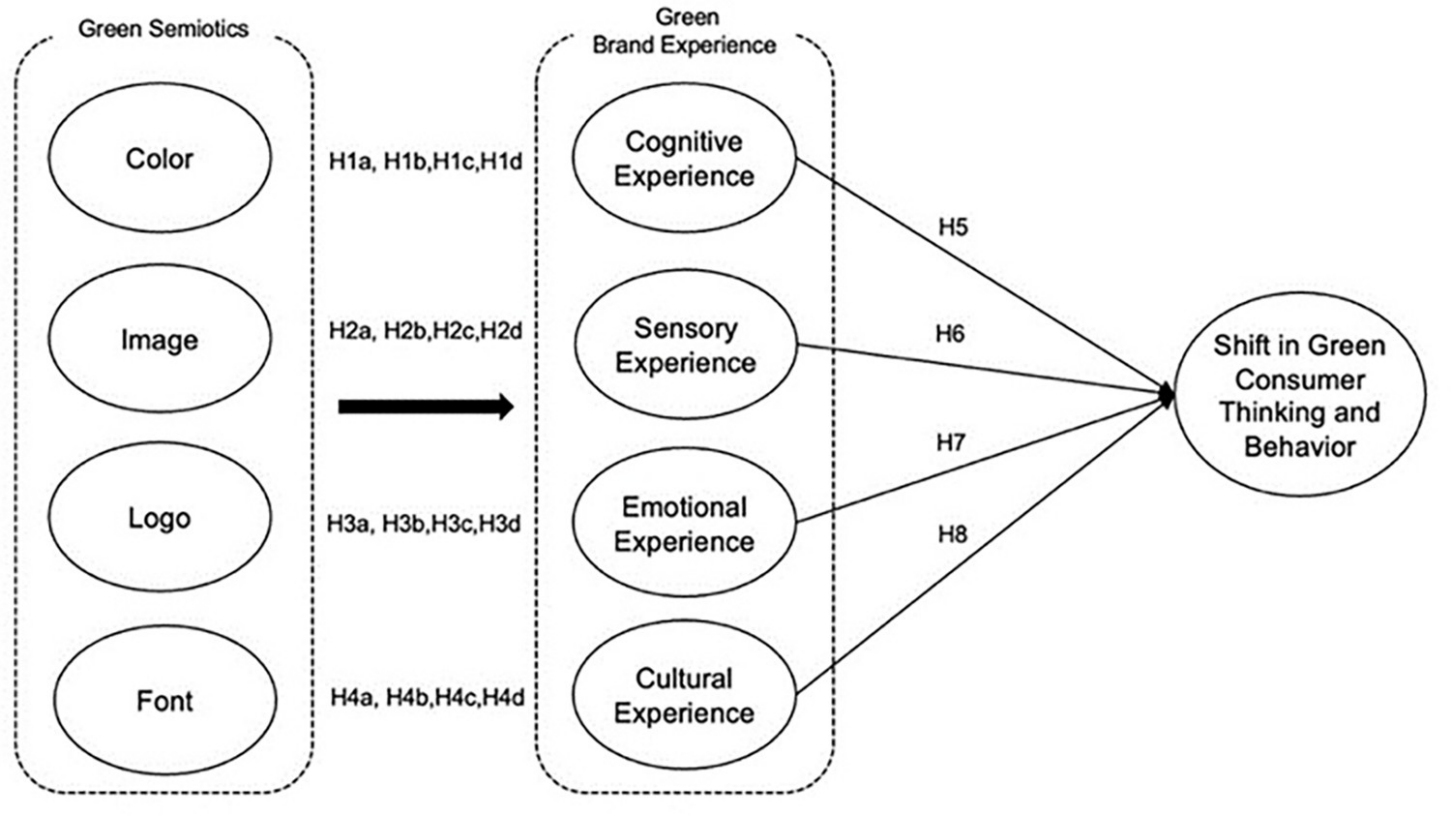
Conceptual framework: Authors, 2024.

The profound impact of Green Brand Experience (GBE) on consumers’ pro-environmental behavioral intentions is critical in understanding the dynamics of green consumerism. GBE encompasses various dimensions such as cognitive, sensory, emotional, and cultural experiences that collectively influence the shift in Green Thinking and Behavior (SGTB) [35] mentioned in Fig 6. Studies have shown that perceived brand innovativeness and a strong green brand image enhance the overall brand experience, which in turn moderates the relationship between GBE and consumer satisfaction [77, 78]. This relationship is further enriched by elements like brand image, trust, value, and loyalty, all contributing to the development of green brand equity and underscoring the vital role of SGTB in advancing sustainability efforts [115]. First, the cognitive dimension of GBE plays a pivotal role, where insights from green semiotics significantly influence green consumption behaviors and thought processes. Factors such as psychological locus of control, green consumption attitudes, and metacognitive experiences are instrumental in shaping these behaviors [116, 117]. Availability of green products also moderates the effects of cognitive factors on consumer behavior [118], while perceptions of green brand quality and satisfaction can drive brand switching behaviors [67]. Second, sensory experiences also play a significant role in reinforcing environmentally friendly behaviors. Positive sensory interactions with green products enhance satisfaction and help sustain green behaviors over time [119]. The interplay between green values and environmental knowledge particularly affects the relationship between green attitudes and behaviors, suggesting that sensory experiences, combined with a deep understanding of environmental issues, can propel green consumer actions [35]. Third, emotionally, the connection with green products is crucial. Research indicates that positive emotional experiences related to green products enhance consumers’ perceptions of green value and their sense of environmental responsibility, ultimately influencing purchase decisions [88, 120]. The emotional domain is especially potent in differentiating the cognitive effort involved in choosing self-interested versus other-interested green products, with the latter often evoking stronger emotional responses [68].Lastly, culturally, the influence of green practices varies significantly across different contexts. Studies have demonstrated the positive effects of green culture on environmentally friendly behaviors in diverse settings, indicating that cultural norms and values can significantly influence green actions independently of economic development factors [121, 122]. In regions where religious and cultural beliefs predominate, such as in Malaysia, Nigeria, Pakistan, and Thailand, green cultural norms are particularly influential in shaping consumer behaviors toward sustainability [48, 123]. Overall, the multidimensional aspects of GBE—cognitive, emotional, sensory, and cultural—play indispensable roles in fostering pro-environmental intentions and behaviors among consumers. These insights underscore the necessity of a holistic approach to green marketing and product design, emphasizing the importance of each dimension in promoting sustainable consumer actions and enhancing the overall effectiveness of environmental sustainability initiatives.

*H5*: *Cognitive experience has positive influence to green behavior and thinking*
*H6*: *Sensory experience has positive influence to green behavior and thinking*
*H7*: *Emotional experience has positive influence to green behavior and thinking*
*H8*: *Cultural experience has positive influence to green behavior and thinking*

## 3. Methodology

This section discusses two important components of research methodology: data collection and sampling, and variable operationalization. This study is conducted using a deductive strategy which follow Ref. [124], which involves deriving hypotheses from the literature review. The research is explanatory since it tests a hypothesis using existing literature and theoretical grounds, rather than exploring new concepts. Data is gathered via surveys using questionnaires and then analyzed using appropriate statistical and mathematical methods to get findings. This approach is particularly suitable for determining connections among many variables.

### 3.1 The ethics statement

This study gathered data from Thai respondents using a quantitative survey questionnaire. Respondents have received, read, and kept a copy of the information letter/plain language statement. Also, respondents have had the opportunity to ask questions about this research and are allowed to withdraw anytime. They understand the general purposes, risks and methods of this research. This study has been reviewed and approved by the Khon Kaen University Ethics Committee in Human Research based on the declaration of Helsinki guidelines under the ethics approval no: HE673016.

### 3.2 Data collection and sampling

This study gathered data from Thai respondents using a quantitative survey questionnaire. A non-probability sampling method with purposive sampling technique is chosen for the data collection process [125]. This method can be particularly useful when studying specifically about customers being green and perception because this method allows researchers to select participants who have specific characteristics or experiences that are relevant to the research question. The potential respondents were selected with some inclusion criteria:

- In adherence to ethical considerations, participants must be over 18 years old.
- Additionally, participants were required to have a comprehension of the concept of "Green products" and acknowledge being influenced by green brands. This requirement was explicitly communicated on the initial page of the online survey, where individuals lacking an understanding of the term "Green products" or not being influenced by the products were politely discouraged from participating.

The respondents were selected based on their purchasing behavior and experience of green products at a minimum in the last three months. The data were obtained from questionnaires by Google Form and Quick Response code (QR code) that were distributed online through e-mail, Instagram direct message, Facebook community, and LINE group. The survey collected responses from representatives in all geographical areas of Thailand, namely: North, Central, North-Eastern, and Southern regions. The survey instrument was split into two sections. The first section includes demographic questions, while the second section comprises the scale items utilized for assessing customers’ perceptions. Based on Cochran calculation [126], the sample size for the survey was calculated using a formula adapted for scenarios where the total population size is unknown. This approach is often necessary in broader studies where the exact population cannot be easily defined.

The formula with the unknown population correction is:

$$n=\frac{z^{2}*\hat{p}(1-\hat{p})}{\varepsilon^{2}}$$

where *z* is 1.96, reflecting a 5% margin of error for a two-tailed distribution (which allocates a margin of 2.5% at each end of the distribution curve). The value of $\hat{p}$, or the estimated proportion of the attribute present in the population, was set at 50% or 0.50. This percentage is commonly used when prior research does not provide a clear expectation, as it maximizes variability, thus providing a conservative estimate. The margin of error *ε* was set at 5%, or 0.05 in decimal form. Applying these parameters, based on Cochran’s formula, the required sample size was calculated to be 385. Out of 400 distributed questionnaires, 385 were returned, but only 357 responses were deemed usable, resulting in a qualification rate of 92% according to source [127], reflecting a high level of data integrity and usability for analysis.

### 3.3 Measure and variable

The study employed previously validated measures, with adjustments made to align with sustainability. **S1 Appendix** contains the modified questionnaire items related to green semiotics and green brand experience. After translation and back-translation into Thai by different bilingual experts, discrepancies were resolved to ensure accuracy. A pilot test involving 40 participants from the target population was conducted to assess clarity, yielding a Cronbach’s alpha value of 0.912, exceeding the threshold [128]. This initial step provided valuable feedback on questionnaire content and structure, guiding revisions. To maintain research integrity, the 40 pilot respondents were excluded from the main study to prevent bias. All constructs were assessed using a five-point Likert scale, with three sets of scales: agreement, anticipation, and tendency, ranging from ‘1′ for ‘strongly disagree’ to ‘5′ for ‘strongly agree.’

Semiotics in green product, or Green Semiotics (GS), involves examining signs, symbols, and communication techniques, including Green Semiotics Color (GSC), Green Semiotics Image (GSM), Green Semiotics Logo (GSL), and Green Semiotics Font (GSF), to convey messages supporting environmental sustainability and eco-friendliness [40, 53]. The first communication technique, Green Semiotics Color (GC), refering to [27, 31, 88] emphasize the utilization and understanding of colors in green semiotics to communicate signals about environmental sustainability design and eco-friendly perception, with factors such as eye-catching design (GC1), combinations of attractive colors (GC2), pop-up and contrast (GC3), color patterns (GC4), and color relativity (GC5). Secondly, Green Semiotics Image (GM) involves the intentional utilization of pictures within the context of green semiotics to convey ideas related to environmental sustainability, conservation, and eco-friendly efforts, including indicators from [6, 53, 94], such as incorporating environmental imagery (GM1), performing charts or illustrations (GM2), and incorporating images of ingredients (GM3). Thirdly, Green Semiotics Logo (GL) involves designing and using logos and symbols to represent and convey messages about environmental sustainability, eco-consciousness, and green activities within the field of green semiotics, with indicators such as utilizing an environmentally themed logo (GL1), incorporating environmental elements like leaves or trees (GL2), and associating with sustainability (GL3) from [100, 102, 104]. The last technique, Green Semiotics Font (GF), refers to the deliberate choice and use of typography in the realm of green semiotics, highlighting the way various typefaces are used to communicate messages related to environmental sustainability, eco-awareness, and green projects, including indicators from [96, 108, 111]such as using fancy fonts reducing attraction (GF1), using bold fonts in design (GF2), and relating font design (GF3).

Green Brand Experience (GBE) encompasses cognitive experience (CE), sensory experience (SE), emotional experience (EE), and cultural experience (CUE) dimensions, representing customers’ entire interaction with a brand’s environmentally friendly efforts, products, and communication, fostering positive associations with environmental sustainability and eco-awareness [40, 106]. Cognitive experience (CE) pertains to individuals’ perceptions, beliefs, and understanding regarding environmental sustainability [13, 70, 84], including aspects such as environmental memories and concern (CE1), cultivation of interest and curiosity (CE2), enhancement of green and environmental cognition (CE3), and recognition of environmentally friendly thinking (CE4). Sensory experience (SE) refers to the sensory perceptions and sensations elicited by interactions with environmentally sustainable products, incorporating elements like sight, smell, touch, and sound, influencing consumers’ overall experience and perception of eco-friendliness through stimuli like sensory stimulation (SE1), attraction to environmental sensory experiences (SE2), and enhancement of eco-sensory experiences (SE3) from [66, 112, 129]. Emotional experience (EE) involves individuals’ emotional reactions when engaging with environmentally friendly initiatives, products, or messages, including emotions like satisfaction, empathy, and concern, shaping their perception and relationship with environmental sustainability and eco-consciousness through factors like provision of green concern (EE1), enhancement of green responsibility (EE2), and mitigation of empathy (EE3) from [88, 114]. Cultural experience (CUE) encompasses individuals’ interactions, perceptions, and behaviors influenced by environmental sustainability and eco-consciousness within their cultural backgrounds, including attitudes, values, traditions, and norms associated with environmental stewardship and sustainable living practices, as indicated by stimuli like attitudes towards green activities (CUE1), reduction of apathetic practices (CUE2), and cultivation of a perception of green culture (CUE3) from [48, 56].

A shift in Green thinking and behavior (SGTB) denotes a change or transformation in individuals’ attitudes, beliefs, and actions towards environmental sustainability and eco-consciousness, reflecting an increased awareness, adoption, and promotion of practices that prioritize the protection and preservation of the environment. This includes indicators such as practicing the 3Rs (SGTB1), concern for environmental impact (SGTB2), engagement in green activities as part of daily routine (SGTB3), and efforts to reduce environmental impact (SGTB4) from [2, 74, 120].

### 3.4 Data analysis

In this study, covariance-based structural squation modeling (CB-SEM), a statistical method designed to analyze complex relationships among variables, was employed following [130–132]. Data analysis was conducted using SPSS and AMOS version 28 and involved several critical steps [133]. Firstly, a descriptive analysis was performed to provide a detailed demographic profile of the sample. Subsequently, the Harman single-factor test was applied, confirming the absence of common method bias. Thirdly, confirmatory factor analysis (CFA) followed, assessing construct validity and reliability; it demonstrated satisfactory factor loadings, Average Variance Extracted (AVE), composite reliability (CR), and Cronbach’s Alpha. Discriminant validity was then established using the Fornell-Larcker criterion and HTMT ratios. Finally, the model’s fit was evaluated using various indices including the Chi-square test, CMIN/DF ratio, Tucker-Lewis Index (TLI), Incremental Fit Index (IFI), Comparative Fit Index (CFI), Root Mean Square Error of Approximation (RMSEA), and Standardized Root Mean Square Residual (SRMR), to ensuring comprehensive validation of the theoretical framework.

## 4. Empirical analysis and results

Covariance-based structural equation modeling (CB-SEM) is a statistical method used to analyze complex relationships among variables [130–132], such as Green Semiotics (GS) elements (color, image, logo, and font) and Green Brand Experience (GBE) dimensions (cognitive, sensory, emotional, and cultural). This analysis, conducted with SPSS AMOS version 28 and SPSS Statistics 28, aims to understand how these variables collectively influence the Shift in Green Thinking and Behavior (SGTB). Given the intricate interplay among these dimensions and elements, SEM allows researchers to identify key drivers of consumer behavior conversion and their interactions within a theoretical framework. This study proceeded through several procedural steps.

Frist step: A descriptive analysis was performed. **Table 1** represents sample demographic. The table presents a comprehensive profile of the surveyed population, revealing a predominant female representation (58.54%) and a majority aged between 20 to 30 years (43.41%). Educationally, most hold bachelor’s degrees (48.74%), and occupationally, the sample comprises business owners (31.37%), private sector employees (29.97%), and public sector workers (19.89%). Income-wise, earners between 30,001 to 40,000 units monthly constitute 21.01% of respondents. Geographically, the central region shows the highest representation (33.05%). Purchasing habits indicate 33.89% buy green products more than four times, underscoring varied consumer behavior in green product consumption.

**Table 1:** Sample demographic.

Characteristic	Category	Total (n = 357)	Percentage (%)
Gender	Male	148	41.46%
	Female	209	58.54%
Age	20–30	155	43.41%
	31–40	123	34.45%
	41–50	65	18.20%
	51–60	14	3.92%
Education	Professional Certificate/Diploma	20	5.60%
	Bachelor’s degree or equivalent	174	48.74%
	Master’s degree	110	30.81%
	Doctoral degree	53	14.85%
Occupation	Student	66	18.49%
	Civil servants/employees in the public sector	71	19.89%
	Private sector employees	107	29.97%
	Business owner	112	31.37%
	Other	1	0.28%
Income (per month)	Under 10,000	33	9.24%
	10,001–20,000	46	12.89%
	20,001–30,000	72	20.17%
	30,001–40,000	75	21.01%
	40,001–50,000	57	15.97%
	More than 50,000	74	20.73%
Region	North	81	22.69%
	Northeast	94	26.33%
	Central region	118	33.05%
	South	64	17.93%
Frequency of purchasing green products	Less than 1 time	26	7.28%
	1–2 times	110	30.81%
	3–4 times	100	28.01%
	More than 4 times	121	33.89%

Secend Step: The Harman single-factor test was conducted to assess the prevalence of a dominant factor within the dataset. The findings indicated that the cumulative variance explained by a single factor in this test was 43.52%, which did not meet the recommended threshold of 50% [134, 135]. Consequently, it is plausible to conclude that the data being analyzed does not exhibit any signs of common method bias.

Third Step: Starting with factor loading, in **Table 2**, confirmatory factor analysis (CFA) was employed to assess the construct validity and reliability of the reflective measurement models estimated by covariance-based SEM [136]. The analysis revealed factor loadings ranging from 0.619 to 0.938, surpassing the recommended threshold of 0.6 or 60% [137]. Additionally, the Average Variance Extracted (AVE) values, ranging from 0.517 to 0.683, exceeded the recommended threshold of 0.5 or 50% of variance [138], indicating convergence among indicators in explaining their variance. Composite reliability (CR) values, ranging from 0.752 to 0.852, were considered adequate, surpassing the cutoff of 0.7 or 70% [133] for measuring the association between indicators measuring the same construct, demonstrating internal consistency and reliability. Cronbach’s Alpha (α) values, ranging from 0.703 to 0.786, indicated a high level of internal consistency within the scale [128]. Furthermore, the variance inflation factor (VIF) values were below commonly used thresholds of 5 or 10, indicating minimal influence of multicollinearity on the variance of coefficients in the model for most variables, with a VIF in the range of 1.152 to 1.946, indicating concerns regarding multicollinearity [136]. This comprehensive analysis provides valuable insights into model robustness, construct validity, and potential issues such as multicollinearity, which are relevant to analyses of consumer behavior and perceptions, offering researchers valuable insights into the implications of their findings.

**Table 2:** Convergent validity and constract reliability.

Construct	Indicator	Factor Loading	AVE	CR	Cronbach’s alpha	VIF
Color	GC1	0.764				1.645
	GC2	0.662				1.483
	GC3	0.734				1.259
	GC4	0.759				1.552
	GC5	0.670	0.517	0.842	0.773	1.457
Image	GM1	0.797				1.493
	GM2	0.792				1.486
	GM3	0.826	0.647	0.846	0.730	1.367
Logo	GL1	0.539				1.188
	GL2	0.619				1.228
	GL3	0.938	0.518	0.752	0.765	1.220
Font	GF1	0.626				1.145
	GF2	0.864				1.524
	GF3	0.821	0.604	0.818	0.759	1.545
Cognitive Experience	CE1	0.782				1.664
	CE2	0.773				1.361
	CE3	0.762				1.587
	CE4	0.753	0.589	0.851	0.769	1.466
Sensory Experience	SE1	0.529				1.152
	SE2	0.928				1.352
	SE3	0.728	0.554	0.780	0.743	1.333
Emotional Experiece	EE1	0.923				1.451
	EE2	0.621				1.267
	EE3	0.760	0.605	0.818	0.703	1.475
Cultural Experince	CUE1	0.927				1.469
	CUE2	0.701				1.468
	CUE3	0.746	0.683	0.838	0.749	1.607
Shifted in Green Behavior and Thinking	SGBT1	0.814				1.371
	SGBT2	0.761				1.512
	SGBT3	0.702				1.842
	SGBT4	0.796	0.591	0.852	0.786	1.946

Fourth Step: Based on empirical evidence, **Table 3** presents the evaluation of discriminant validity, assessing the distinctiveness of each construct within the structural model. The Fornell-Larcker criterion is a widely recognized method for assessing the discriminant validity of measurement models. Fornell and Larcker [139] assert that to establish discriminant validity, the square root of the Average Variance Extracted (AVE) for any given construct—represented on the diagonal of the correlation matrix—must exceed the correlations between that construct and all other constructs within the model. This requirement ensures that a construct is more strongly associated with its own indicators than with those of any other construct, thereby affirming the distinctiveness of the constructs in the measurement model. Moreover, the HTMT values observed in the study fell within the range of 0.108 to 0.763, satisfying the criterion proposed by [140], which suggests values below 0.90 to indicate adequate discriminant validity. Consequently, it can be inferred that multicollinearity was not a concern, affirming the presence of discriminant validity within the model.

**Table 3:** Discriminant validity.

Fornell-Larcker Criterion									
	CE	SGTB	CUE	EE	SE	GC	GF	GL	GM
**CE**	**0.767**								
**SGBT**	0.165	**0.769**							
**CUE**	0.058	-0.047	**0.826**						
**EE**	-0.094	-0.243	-0.464	**0.778**					
**SE**	-0.146	-0.32	-0.472	0.708	**0.744**				
**GC**	0.074	0.213	0.293	-0.234	-0.275	**0.719**			
**GF**	0.273	0.129	0.152	-0.190	-0.200	0.368	**0.777**		
**GL**	-0.131	-0.139	-0.65	0.718	0.739	-0.276	-0.189	**0.720**	
**Heterotrait-Monotrait ratio of correlations: HTMT**									
**CE**									
**SGBT**	0.190								
**CUE**	0.108	0.133							
**EE**	0.117	0.268	0.43						
**SE**	0.206	0.451	0.553	0.705					
**GC**	0.134	0.231	0.327	0.236	0.341				
**GF**	0.346	0.194	0.19	0.256	0.257	0.533			
**GL**	0.173	0.191	0.62	0.742	0.749	0.279	0.248		
**GM**	0.349	0.17	0.394	0.282	0.312	0.688	0.763	0.241	

Fift Step: The examination of the measurement model reveals positive outcomes in **Table 4**, supported by various fit indices. The chi-square value, significant at a p-value of less than 0.05, along with a Chi-square/degrees of freedom (CMIN/DF) ratio of 2.073, indicates a reasonable fit between the theoretical model and observed data [141]. Moreover, the Tucker-Lewis Index (TLI), Incremental Fit Index (IFI), and Comparative Fit Index (CFI) surpass the suggested threshold of 0.9 [142], demonstrating strong correspondence between the proposed model and the data (TLI = 0.955, CFI = 0.955, IFI = 0.960 for the measurement model). Additionally, Root Mean Square Error of Approximation (RMSEA) and Standardized Root Mean Square Residual (SRMR) values remain within acceptable ranges. Upon transitioning to the structural model, while the CMIN/DF ratio increases slightly, it remains below the criterion of 3 [141], and key fit indices remain above 0.9 [142] (TLI = 0.914, CFI = 0.968, IFI = 0.972 for the structural model). These findings suggest a robust model fit, although researchers should consider contextual nuances and model complexities when interpreting the results.

**Table 4:** Model fit indices for measurement model and structural model.

Model fit measure indices	Measurement Model	Structural Model	Threshold values
Chi-square	274.456 (p < 0.001)	326.542 (p < 0.001)	p < 0.05
CMIN/DF	2.073	1.669	Less than 3
TLI	0.925	0.914	> 0.9
CFI	0.955	0.968	> 0.9
IFI	0.960	0.972	> 0.9
RMSEA	0.063	0.051	< 0.08
SRMR	0.045	0.048	< 0.08

Note: CFI = Comparative Fit Index, TLI = Tucker-Lewis Index, SRMR = Standardized Root Mean Square Residual, RMSEA = The Root Mean Square Error of Approximation CMIN/DF = The Chi-square/degrees of freedom ratio, IFI = Incremental Fit Index

### 4.1 Hypothesis testing

The research employs a standard error-based method to analyze data and elucidate the connections between green semiotics, dimensions of green brand experience, and the shift in consumer attitudes and behaviors towards eco-friendly products. The primary aim is to investigate the fundamental components constituting green semiotics and dimensions of green brand experience inherent in eco-friendly products. Furthermore, the study delves into the interaction between semiotics and brand experience dimensions of eco-friendly products, seeking to comprehend their combined influence on shaping consumer attitudes and behaviors towards green products. In the contemporary global landscape, semiotic packaging proves advantageous as it enables marketers to convey brand experiences and interpretations across diverse cultures more effectively. Utilizing related images, content, and graphics, brand semiotics on product packaging evoke sensory impressions, foster personal connections, and prompt customers to engage with the brand on a deeper level. Additionally, previous literature has illustrated how brand experience dimensions contribute to the transformation of consumer attitudes and behaviors, ultimately promoting the adoption of eco-friendly practices and products.

In this comprehensive analysis, we investigated the relationships between green semiotics elements and brand experience dimensions to discern how these elements affect consumer perceptions and behaviors towards eco-friendly products, as presented in Table 5. The green semiotics were operationalized into four main components: Green Semiotic Color (GC), Green Semiotics Image (GM), Green Semiotics Logo (GL), and Green Semiotics Font (GF). Each was analyzed for its impact on different facets of brand experience: Cognitive Experience (CE), Sensory Experience (SE), Emotional Experience (EE), and Cultural Experience (CUE).

**Table 5:** Hypothesis test table.

H	Path Relationships	Standardized Estimated (β)	Standard Errors	T-values	P-values	Predicted Effect	Observed Effect	Decision
H1a	GC -> CE	-0.11	0.085	1.265	0.207	Positive	Null	Rejected
H1b	GC -> SE	-0.25	0.040	3.613	***	Positive	Negative	Contradicted
H1c	GC -> EE	-0.265	0.127	3.542	***	Positive	Negative	Contradicted
H1d	GC -> CUE	0.236	0.111	3.367	***	Positive	Positive	Supported
H2a	GM -> CE	0.279	0.112	2.735	**	Positive	Positive	Supported
H2b	GM -> SE	-0.483	0.079	3.979	***	Positive	Negative	Contradicted
H2c	GM -> EE	-0.468	0.243	3.693	***	Positive	Negative	Contradicted
H2d	GM -> CUE	0.455	0.195	4.165	***	Positive	Positive	Supported
H3a	GL -> CE	-0.018	0.112	0.235	0.814	Positive	Null	Rejected
H3b	GL -> SE	0.776	0.136	5.078	***	Positive	Positive	Supported
H3c	GL -> EE	0.766	0.429	4.652	***	Positive	Positive	Supported
H3d	GL -> CUE	-0.551	0.315	4.255	***	Positive	Negative	Contradicted
H4a	GF -> CE	0.278	0.462	1.339	0.182	Positive	Null	Rejected
H4b	GF -> SE	-0.095	0.091	1.381	0.168	Positive	Null	Rejected
H4c	GF -> EE	-0.107	0.300	1.388	0.166	Positive	Null	Rejected
H4d	GF -> CUE	0.037	0.243	0.551	0.582	Positive	Null	Rejected
H5	CE -> SGBT	0.137	0.082	1.881	0.061	Positive	Null	Rejected
H6	SE -> SGBT	-0.288	0.046	11.84	***	Positive	Negative	Contradicted
H7	EE -> SGBT	-0.217	0.071	1.984	*	Positive	Negative	Contradicted
H8	CUE -> SGBT	-0.251	0.065	2.686	**	Positive	Negative	Contradicted

**Note:** * Significant at < 0.05, **Significant at < 0.01, ***Significant at < 0.001.

The empirical findings from the analysis led to mixed results across various hypotheses. Starting with **H1**, which proposed a generally positive relationship between Green Semiotic Color (GC) and the dimensions of brand experience, the results varied. Specifically, GC did not significantly influence Cognitive Experience (CE) as predicted, showing a nonsignificant negative beta (H1a; β = -0.11) with a p-value of 0.207, suggesting a lack of support for this positive relationship. In contrast, GC had a significantly negative impact on both Sensory (H1b; β = -0.250, p < 0.001) and Emotional Experiences (H1c; β = -0.265, p < 0.001), leading to the rejection of the positive influence hypothesized. Interestingly, GC positively affected Cultural Experience (H1d; β = 0.236, p < 0.001), which aligned with the initial predictions.

**H2** explored the influence of Green Semiotics Image (GM) on brand experiences. The results were supportive for Cognitive (H2a; β = 0.279, p = 0.007) and Cultural Experiences (H2d; β = 0.455, p < 0.001), indicating that GM positively impacts these dimensions. However, contrary to expectations, GM had a detrimental effect on Sensory (H2b; β = -0.483, p < 0.001) and Emotional Experiences (H2c; β = -0.468, p < 0.001), contradicting the hypothesized positive effects.

For **H3**, which pertained to Green Semiotics Logo (GL), the findings were favorable for Sensory (H3b; β = 0.776, p < 0.001) and Emotional Experiences (H3c; β = 0.766, p < 0.001), supporting the hypothesis of a positive relationship. However, GL negatively influenced Cultural Experience (H3d; β = -0.551, p < 0.001) and showed no significant effect on Cognitive Experience (H3a; β = -0.018, p = 0.814), thus not supporting those aspects of the hypothesis.

**H4**, examining Green Semiotics Font (GF), found no significant impact on any brand experience dimensions (H4a; β = 0.278, p = 0.182; H4b; β = -0.095, p = 0.168; H4c; β = -0.107, p = 0.166; H4d; β = 0.037, p = 0.582), rejecting all sub-hypotheses related to GF.

Additional analyses assessed the effect of brand experience dimensions on Green Behavior and Thinking (SGBT). Cognitive Experience (CE) showed a non-significant positive effect on SGBT (**H5**; β = 0.137, p = 0.061), and significant negative impacts were observed for Sensory (**H6**; β = -0.288, p < 0.001), Emotional (**H7**; β = -0.217, p = 0.048), and Cultural Experiences (**H8**; β = -0.251, p = 0.008) contrary to the predicted positive influences.

These findings illustrate a complex landscape where green semiotics variously influence consumer responses, with several instances where expected positive impacts were instead negative, underscoring the intricate dynamics at play in eco-friendly product branding.

### 4.2 Mediation analysis

This research delves into the intricate relationship between green semiotics and consumer behavior by expanding on the mediation analysis framework developed by [144] and using sophisticated analytical methods offered by [145]. Our results align with the intricate mediation patterns outlined in other studies [100, 119], demonstrating that green semiotics components have a substantial impact on customer perceptions and actions [32]. Furthermore, the conflicting mediation effects that were discovered highlight the complex psychological experiences involved, a phenomenon that has been previously acknowledged in environmental psychology [64, 89] and consumer behavior research [53, 118]. This research adds to the developing knowledge of how green symbols might impact green consumer behavior, emphasizing the need for further investigation in this field [19].

Mediation analysis serves as a statistical technique that allows for the exploration of how an intermediate variable, known as the mediator, may account for the link between an independent and a dependent variable. It aims to delineate the mediator’s direct and indirect contributions, as well as its aggregate influence on the primary relationship under investigation. The outcomes of this mediation analysis are detailed in Table 6. A commonly utilized interpretive measure in mediation analysis is the Variance Accounted For (VAF). In the given analysis, the VAF values suggest a degree of partial mediation in complete form, which clarify by [143], ranging from 38.50% to 56.69%. Such a VAF range, falling between 20% and 80%, is indicative of partial mediation. These findings suggest a significant mediating role of Sensory and Emotional Experience between the independent variables—Green Semiotics Color, Logo, and Image—and the dependent variable, Green Behavior and Thinking. In addition, a scenario where the VAF exceeds 80% points to full mediation, observed in the relationship where Green Semiotics Color as the independent variable affects Green Behavior and Thinking as the dependent variable, through Cultural Experience as the mediator.

**Table 6:** Mediation analysis.

Mediation relationships	a	b	c	Indirect effect (a x b)	Total effect (a x b) + c	VAF	Mediation Results [143]
GC -> CE -> SGTB	0.279 (n.s.)	0.137(n.s.)	0.055 (n.s.)	N.A.	N.A.	N.A.	No Mediation	Indepentdent Model
GC -> SE -> SGTB	-0.25 ***	-0.288***	0.055 (n.s.)	0.072	0.127	56.69%	Partial Mediation	Complete Mediation
GC -> EE -> SGTB	-0.265***	-0.217*	0.055 (n.s.)	0.058	0.113	51.33%	Partial Mediation	Complete Mediation
GC -> CUE -> SGTB	0.236***	-0.251**	0.055 (n.s.)	N.A.	N.A.	N.A.^+^	Full Mediation	Complete Mediation
GM -> CE -> SGTB	0.279**	0.137(n.s.)	0.164 (n.s.)	0.038	0.202	18.81%	No Mediation	Single Effect
GM -> SE -> SGTB	-0.483***	-0.288***	0.164 (n.s.)	0.139	0.303	45.87%	Partial Mediation	Complete Mediation
GM -> EE -> SGTB	-0.468***	-0.217*	0.164 (n.s.)	0.102	0.266	38.35%	Partial Mediation	Complete Mediation
GM -> CUE -> SGTB	-0.018***	-0.251**	0.164 (n.s.)	N.A.	N.A.	N.A.	No Mediation	Complete Mediation
GL -> CE -> SGTB	-0.018 (n.s.)	0.137(n.s.)	-0.253 (n.s.)	N.A.	N.A.	N.A.	No Mediation	Indepentdent Model
GL -> SE -> SGTB	0.776***	-0.288***	-0.253 (n.s.)	N.A.	N.A.	N.A.	Partial Mediation	Complete Mediation
GL -> EE -> SGTB	0.766***	-0.217*	-0.253 (n.s.)	N.A.	N.A.	N.A.	Partial Mediation	Complete Mediation
GL -> CUE -> SGTB	-0.551***	-0.251**	-0.253 (n.s.)	N.A.	N.A.	N.A.	No Mediation	Complete Mediation
GF -> CE -> SGTB	0.278(n.s.)	0.137(n.s.)	0.08 (n.s.)	0.038	0.118	32.20%	Partial Mediation	Indepentdent Model
GF -> SE -> SGTB	-0.095 (n.s.)	-0.288***	0.08 (n.s.)	0.027	0.107	25.23%	Partial Mediation	Single Effect
GF -> EE -> SGTB	-0.107 (n.s.)	-0.217*	0.08 (n.s.)	0.023	0.103	22.33%	Partial Mediation	Single Effect
GF -> CUE -> SGTB	0.037 (n.s.)	-0.251**	0.08 (n.s.)	N.A.	N.A.	N.A.	No Mediation	Single Effect

**Note:** p-value <0.1*; p-value <0.05*; p-value <0.01**; p-value <0.001***; n.s. = p-value is not significant; N.A. = not applicable (with negative value)., + value more than 100%

Subsequent to the affirmation of the hypotheses put forward, the ensuing section will undertake a comparative analysis with antecedent studies, aiming to scrutinize both the alignment and the variances presented by the current research outcomes.

## 5. Discussions

This study aims to investigate the core elements of green semiotics—color, image, logo, and font—and their impact on the dimensions of green brand experience inherent in eco-friendly products, specifically assessing cognitive, sensory, emotional, and cultural experiences, and how green brand experience influences green customer thinking and behavior. The empirical results indicate that most relationships between green semiotics elements and the dimensions of green brand experience, as well as the interactions within these dimensions, are significantly negative, highlighting the complex influence these elements exert on consumer perceptions and behaviors.

Examining **H1**, we observed that green semiotic color (GC) did not significantly influence cognitive experience (CE), **H1a**, presenting a non-significant negative beta value. This result suggests a disconnect between the anticipated positive influence of GC and the actual perceptions of Thai consumers, which against [55], potentially indicating a skepticism towards the use of color in green marketing. Such skepticism may stem from concerns that the colors employed do not genuinely represent sustainable efforts, but rather serve as superficial embellishments—often labeled as “greenwashing” [146]. Furthermore, the detrimental impact of GC on sensory and emotional experiences suggests that over-saturated colors or inappropriate application may provoke adverse sensory and emotional responses, which is contradicted the **H1b** and **H1c**. This finding is unexpected, particularly in light of previous research, [82, 85], indicating a positive correlation between color and environmental sensory and emotional experiences. The apparent lack of influence of color in evoking clear perceptions may distort the relationship between sensory and emotional experiences, leading to a misalignment with the brand and its image [147, 148]. Interestingly, GC positively affects cultural experiences, which is supported **H1d**, supporting the notion that when colors are thoughtfully aligned with cultural values and environmental expectations, they can enhance the green brand experience, as same as previous study of [56]. This enhancement occurs through the communication of new environmental values where the colors themselves may also reflect the organization’s green culture [121]. This dynamic underscores the importance of strategically selecting colors that resonate with cultural and environmental norms to reinforce the authenticity and integrity of green marketing efforts.

In the Thai context, the investigation into **H2** revealed that while images in green product packaging significantly enhance cognitive understanding and cultural connection to the product, **H2a** and **H2d** supported by [27, 99]. However, images unexpectedly negatively impact sensory and emotional experiences, **H2b** and **H2c** contrasted inconsistently [66, 88]. This discrepancy arises when imagery sets unrealistic expectations or fails to align with the actual product, leading to disillusionment and emotional disconnect [149]. For instance, idealized natural imagery that does not accurately depict the product’s features may create a sensory gap between anticipated and actual experiences, eliciting negative reactions and potentially undermining brand loyalty [150] These findings underscore the necessity for marketers to use authentic visual representations that accurately communicate the ecological benefits of the product, avoiding over-promising which can contribute to skepticism and accusations of greenwashing [27, 91, 93, 100]. This approach is crucial in maintaining trust and enhancing the credibility of green marketing claims in Thailand.

The exploration of **H3** underscores the complex role logos play in shaping consumer perceptions of green product packaging. While logos incorporating eco-friendly themes such as leaves, water, or earth are successful in eliciting positive sensory and emotional responses (**H3b** and **H3c** supported by [49, 102]), and logos influence on cognitive and cultural resonance presents challenges, contrasting with previous studies [56, 100]. Logos effectively foster sensory gratification and emotional connections, enhancing feelings of wellness and environmental stewardship [102], which play pivotal roles in attracting consumers to the product’s eco-friendly qualities [49, 88, 103]. However, they falter in generating a positive cognitive impact, **H3a** rejected due to no significant effect, where the superficial or greenwashed nature of these logos may fail to convince consumers of the brand’s genuine commitment to sustainability [151]. Furthermore, the negative cultural impact (**H3d** contradicted, showing a negative effect) highlights a significant disconnect between the logos and local cultural narratives or environmental values in Thailand. This incongruence can result from logos that do not align with the indigenous cultural values linked to environmentalism, leading to perceptions of inauthenticity and superficiality [152, 153]. Research indicates that such discrepancies can significantly undermine brand equity and consumer loyalty, particularly when logos appear as mere superficial gestures rather than authentic representations of green initiatives [154]. This disconnection can alienate culturally diverse markets, where cultural congruence is crucial for brand credibility. The findings suggest that while logos can successfully appeal to the sensory and emotional dimensions, their failure to resonate culturally and cognitively due to perceived greenwashing necessitates a more nuanced approach. Brands operating in Thailand need to ensure that their logo designs not only appeal to the immediate sensory and emotional aspects but also deeply resonate with the cultural ethos of the local population.

The examination of **H4**, which explored the impact of font selection on the green brand encounter—encompassing cognitive, sensory, emotional, and cultural dimensions—revealed an intriguing finding: font selection does not significantly influence these aspects of brand perception, which is rejected **H4a, H4b, H4c, H4d**. This result challenges the pervious researches that font style plays a critical role in shaping consumer impressions [34, 54, 110, 114]. However, research in visual communication suggests that while font selection can influence text legibility and attractiveness [110], its impact appears to be subdued in green branding scenarios within Thailand. Here, other elements such as color, imagery, and logos tend to have a more pronounced effect in conveying environmental messages [53, 100, 102]. An investigation by [34] supports this observation, indicating that although specific font attributes might influence brand personality perceptions, these effects are often overshadowed by the content and other visual components of the packaging. Moreover, according to [54], the influence of typography on customer experience in green branding may be limited, as the emotional and cognitive responses to typography are nuanced and highly dependent on specific circumstances. The minimal impact observed suggests that Thai consumers prioritize the message and authenticity of environmental claims over the stylistic presentation of the text. This aligns with broader concerns about greenwashing, where superficial environmental claims are scrutinized by consumers who are becoming increasingly aware of and sensitive to the genuineness of such claims.

In the context of Thailand, our investigation into the impact of cognitive, sensory, emotional, and cultural experiences on green thinking and behavior has revealed surprising transformations that can detrimentally affect consumer engagement with environmentally friendly products. While previous study of [67, 84, 129, 155] supported that cognition, sensory appeal, emotional connection, and cultural relevance are generally assumed to bolster customer loyalty towards products. But even so, our findings suggest that these factors can sometimes have adverse effects within the realm of green branding, leading to the rejection of **H5** and contradiction of **H6**, **H7**, and **H8**, all showing negative or null effects. The rejection of the cognitive dimension **(H5)** can be attributed to the pervasive issue of greenwashing, where superficial or misleading claims undermine the credibility of environmental messaging, leading Thai consumers—who are increasingly aware and informed about environmental issues—to scrutinize and often dismiss cognitive appeals in green branding as insincere or manipulative, emphasizing the need for transparency and authenticity in communicating environmental benefits [14, 156]. Previous study indicates that overly artificial sensory attributes, or those that deviate significantly from the natural qualities expected of environmentally friendly products, can foster mistrust or diminish consumer confidence [115]. Emotional appeals that fail to align genuinely with consumer beliefs, or that overly promote environmental benefits, may lead to disenchantment and negatively impact brand perception [123]. Furthermore, cultural misalignments—where green marketing messages do not resonate with local values or appear culturally insensitive—can alienate potential consumers [155], exacerbating the challenges of greenwashing perceptions in the Thai market.

Moreover, the paradoxical outcome in the mediation analysis, particularly within the context of greenwashing, can be further explored. Firstly, the positive influence of Green Semiotic Color (GC) on Cultural Experience (CUE) may be perceived by consumers as a superficial marketing tactic rather than a genuine commitment to sustainability. This skepticism can stem from a history of companies using green-themed marketing to appeal to environmental sensibilities without substantive environmental actions, diluting the trust consumers have in green claims [91]. Secondly, even when GC successfully enhances cultural resonance, if consumers perceive this alignment as merely aesthetic or promotional, their overall confidence in the brand’s environmental claims may wane [103]. This disconnect may be exacerbated by widespread media coverage and consumer awareness of greenwashing practices, making consumers more vigilant and critical of the authenticity behind green marketing efforts [157]. Lastly, the negative shift in Green Behavior and Thinking (SGBT) despite positive cultural influences highlights a critical need for marketers to back up visual and cultural appeals with tangible environmental actions and transparent communication about their sustainability efforts. Ensuring that green marketing strategies are rooted in actual environmental benefits is essential to mitigating the effects of greenwashing and fostering genuine consumer engagement with sustainably marketed products [146].

### 5.1 Implication

#### 5.1.1 Theoretical implication

Semiotic theories and methods can be employed to discern patterns in popular culture, comprehend the development of consumer attitudes and behavior in relation to popular culture, including brands, and optimize marketing and advertising initiatives by enhancing communication with the end user. There are four key theoretical implications proposed to the literature as follows, which significantly advance our understanding of how green semiotics influence consumer perception and behavior within the context of environmental marketing:

1. First, this research contributes to the theoretical understanding of how specific semiotic elements influence green consumer behavior, refining the existing theories on green semiotics. By revealing that the relationship between semiotic elements like color, logo, image, and font and green brand experience dimensions is predominantly negative, the study challenges the conventional wisdom that such elements are universally beneficial in promoting eco-friendly behavior. It suggests that the effectiveness of these elements is more nuanced and dependent on their alignment with consumer expectations and cultural norms.
2. Second, the findings also enrich brand management theory by emphasizing the importance of integrating sensory, emotional, cognitive, and cultural dimensions in the branding strategy of eco-friendly products. The research underscores the need for a holistic approach in green branding that considers all these dimensions to foster positive consumer engagement and avoid the pitfalls of greenwashing that can alienate consumers.
3. Third, this study extends the theoretical discussion on the role of cultural congruence in environmental marketing. By demonstrating that cultural misalignments can negatively affect consumer responses to green marketing efforts, it provides a theoretical basis for the argument that successful green marketing must be culturally sensitive and aligned with local values and practices to be effective.
4. Lastly, the study contributes to behavioral change theories by highlighting the complex pathways through which green brand experiences shape consumer behavior and attitudes toward sustainability. The unexpected negative impacts on green thinking and behavior challenge existing models that posit a straightforward positive influence of brand experiences on behavior change. This invites researchers to explore deeper into the psychological and social factors that mediate these relationships, potentially leading to more comprehensive models that account for both positive and negative influences.

#### 5.1.2 Practical implications

The practical implications of this study are profound, offering valuable insights for businesses engaged in green marketing and sustainability initiatives. These findings can inform and transform current practices by highlighting the importance of aligning green semiotic elements with consumer expectations and cultural values. Here are four key practical implications that emerge from this research, each proposing strategies to enhance the effectiveness and authenticity of green marketing efforts:

1. First, this research provides actionable insights for companies looking to refine their green branding strategies. By identifying the negative impacts of certain semiotic elements on consumer perception, businesses can tailor their branding to avoid potential pitfalls. For example, companies can focus on ensuring that the use of colors, logos, and images in their packaging truly reflects their environmental commitments, thus enhancing authenticity and reducing consumer skepticism about greenwashing.
2. Second, the findings underscore the importance of cultural congruence in marketing communications. Marketers should develop campaigns that are specifically tailored to align with the cultural values and environmental beliefs of their target market. This customization can increase the effectiveness of marketing efforts by resonating more deeply with consumers, leading to improved brand loyalty and advocacy for eco-friendly products [72].
3. Third, given the complex influences of green semiotics on consumer behavior, there is a practical need for businesses to engage in and facilitate more educational outreach. By educating consumers about the sustainability efforts behind their products and how these efforts are represented through various semiotic elements, companies can foster a more informed consumer base that is capable of making more conscious purchasing decisions.
4. Fourth, the unexpected findings regarding the negative impacts of certain green semiotics suggest that companies could benefit from developing new metrics to assess the effectiveness of their green branding efforts. These metrics should not only evaluate the immediate appeal of marketing materials but also consider long-term impacts on consumer trust and loyalty. Establishing such metrics can help businesses track and optimize the real value added by their green marketing strategies, ensuring they contribute positively to both brand perception and environmental goals.

A semiotic analysis involves deciphering the signifiers that evoke specific meanings and resonate with your audience. Armed with this knowledge, companies can integrate the deciphered elements into its brand and across all marketing communications.

Conducting a semiotic analysis can be included in a checklist for the creation of a new advertising campaign or the publication of a central content asset. Companies have the option to conduct the analysis independently or in collaboration with marketing team. Furthermore, consider extending invitations to delegates from other departments or, if financially feasible, individuals from intended demographic.

## 6. Conclusion

This section provides an overview of three fundamental components of the study’s findings, namely the primary conclusion, its limitations, and potential avenues for future research. This study ventured into the uncharted territory of green semiotics and their impact on the green brand experience, specifically examining how elements such as color, image, logo, and font influence consumer perceptions and behaviors towards eco-friendly products. The primary conclusion reveals a complex and often negative relationship between these semiotic elements and the dimensions of green brand experience—cognitive, sensory, emotional, and cultural. This suggests that while eco-friendly brands aim to leverage these elements to foster a positive green image, there may be unintended consequences that could hinder the effectiveness of these marketing strategies.

This study’s focus on the Thai market limits its generalizability to other cultural and environmental contexts, potentially restricting the broader applicability of its findings. The reliance on cross-sectional data to gauge consumer perceptions and behaviors represents a significant constraint, as it captures data at a single moment and overlooks the evolving dynamics of consumer attitudes and the changing nature of brand experiences over time—factors that are particularly relevant in green marketing. Additionally, the use of self-reported measures introduces the risk of bias, potentially compromising the accuracy of the findings. The complex nature of the green semiotics concept also poses challenges in distinguishing the effects of specific elements from the overall marketing mix. To address these limitations, future research should aim to replicate the study across different cultural and environmental settings, incorporate longitudinal designs to track changes in consumer perceptions and behaviors over time, and employ alternative methods beyond self-reported measures, such as behavioral observations or experimental designs. Furthermore, a more granular approach to disentangle the complex interactions within the green semiotics concept is necessary, focusing on isolating specific elements and examining their individual and combined effects. By overcoming these limitations, future research can enhance the generalizability of the findings, provide a richer understanding of green semiotics’ impact on consumer behavior globally, and offer deeper insights into the strategic use of green semiotics in fostering sustainable consumer practices and enhancing green brand perception.

## Supporting information

S1 Appendix The constructs used in the article. (DOCX)
