# Supplementary material for: Unlocking the secrets of green semiotics: The revolutionary power of eco-symbols in transforming consumer perceptions and catalyzing behavioral shifts in emerging markets
Source: PLoS One. 2024 Sep 26;19(9):e0310963. doi: 10.1371/journal.pone.0310963 (PMC11426538; doi:10.1371/journal.pone.0310963)
Supplement: S1 Appendix — (DOCX) [file pone.0310963.s001.docx]

# **Appendix A: The constructs used in the article**

| **Construct** | **Indicators** | **References** |
| --- | --- | --- |
| ***Semiotics of the Green Product*** | |  |
| Color | **GC1.** Catching the first sight by looking at sustainable product package.  **GC2.** Combination of attractive color on green brand attract customers.  **GC3.** Creating visibility of the green product by using pop-up and contrast color**.**  **GC4.** Utilizing the different color pattern enhances differentiate the sustainable product.  **GC5.** Attracting with a blend of color schemes that resonate with green and eco-friendly product themes. | [27,31,88] |
| Image | **GM1.** Establishing a green brand identity by incorporating environmental imagery (e.g., leaf, earth, and plant) on the packaging of eco-friendly products.  **GM2.** Creating green product prefer by showing a chart or illustration on packaging. **GM3.** Incorporating images of the ingredients in green products to showcase transparency. | [6,53,94] |
| Logo | **GL1.** Building a sustainable brand identity by utilizing an environmentally themed logo.  **GL2.** Enhancing the eco-friendly brand image of the product by incorporating environmental elements into the logo.  **GL3.** Building brand value through the use of a logo and/or signage associated with sustainability. | [100,102,104] |
| Font | **GF1.** Using fancy fonts reduces the attraction of green products.  **GF2.** Using bold fonts in design for critical information enhances the understanding of the brand's commitment to green initiatives.  **GF3.** Printing product information in relate font design on the packaging aids in evaluating the green product. | [96,108,111] |
| ***Green Brand Experience*** | |  |
| Cognitive Experience | **Employing green awareness semiotics ....**  **CE1**. to provide environmental memories and concern.  **CE2.** to promote the cultivation of green interest and curiosity.  **CE3.** to enhance green and environmental cognition.  **CE4.** to rise the recognition of environmentally friendly thinking. | [13,70,84] |
| Sensory Experience | **Utilizing green awareness semiotics ...**  **SE1.** to stimulate the sense of taste by incorporating visually appealing images on green product packaging.  **SE2.** to attract the sensory of environmental and green by employing sustainable color themes.  **SE3.** to enhance the eco- sensory by creating green concept logo and slogan. | [66,112,129] |
| Emotional Experience | **Incorporating green awareness semiotics...**  **EE1**. to provide the positive feeling about minimize environmental impart.  **EE2.** to enhance the green feeling connection between customers and brand.  **EE3**. to mitigate negative sentiments associated with waste and environmental impact. | [88,114] |
| Cultural Experience | **Applying green awareness semiotics...**  **CUE1.** to stimuli green attitude toward minimizing environmental impact.  **CUE2**. to reduce apathetic environmental practice, such as improper disposal of plastic bottles and plastic waste.  **CUE3.** to cultivate a perception of a green culture associated with the green brand and its products. | [48,56] |
| Shifted Green thinking and behavior | **I would practice…**  **SGTB1.** 3Rs to minimize waste and environmental impact after using green product.  **SGTB2.** concerning about environmental impact after buying eco-friendly product.  **SGTB3.** changing in everyday lifestyle to be more eco-friendly routine.  **SGTB4.** inhibiting environmental impact activities in the future. | [2,74,120] |
